# *Xanthomonas oryzae* Orphan Response Regulator EmvR Is Involved in Virulence, Extracellular Polysaccharide Production and Cell Motility

**DOI:** 10.1111/mpp.70083

**Published:** 2025-04-06

**Authors:** Pei‐Dong Ren, Zeng‐Feng Ma, Qing‐Qing Liu, Xin‐Qi Xia, Gui‐Ning Zhu, Ji‐Liang Tang, Rui‐Fang Li, Guang‐Tao Lu

**Affiliations:** ^1^ Plant Protection Research Institute, Guangxi Academy of Agricultural Science, Key Laboratory of Green Prevention and Control on Fruits and Vegetables in South China Ministry of Agriculture and Rural Affairs, Guangxi Key Laboratory of Biology for Crop Diseases and Insect Pests Nanning China; ^2^ State Key Laboratory for Conservation and Utilization of Subtropical Agro‐Bioresources, College of Life Science and Technology Guangxi University Nanning China; ^3^ Rice Research Institute, Guangxi Academy of Agricultural Sciences Nanning China

**Keywords:** motility, single‐domain response regulator, two‐component signalling system, type IV pili, *Xanthomonas oryzae*

## Abstract

Bacteria have evolved a large number of two‐component signalling systems (TCSs), which are typically composed of a histidine sensor kinase (HK) and a response regulator (RR), to sense environmental changes and modulate subsequent adaptive responses. Here, we describe the involvement of an orphan single‐domain RR named EmvR in the virulence, extracellular polysaccharide (EPS) production and cell motilities of the bacterial leaf streak pathogen *Xanthomonas oryzae* pv. *oryzicola* (Xoc), which infects rice leaves mainly via stomata and wounds. Deletion of *emvR* in Xoc reduced virulence when using spraying inoculation but not when using infiltration inoculation. The *emvR* deletion mutant displayed weakened spreading and enhanced twitching. Additionally, although deletion of *emvR* did not significantly affect EPS production, overexpression of *emvR* significantly increased EPS production. Several standard assays revealed that EmvR physically interacts with PilB and represses its ATPase activity. Combining our data with previous findings that PilB provides the energy for type IV pilus (T4P) biogenesis, we conclude that EmvR plays a vital role in modulating Xoc T4P synthesis and in the early stage of Xoc infection through rice stomata. Moreover, our data reveal that EmvR can also interact with the HK of the TCS ColS_XOCgx_4036_/ColR_XOCgx_4037_, which positively and negatively affects Xoc spreading and twitching, respectively. We propose a ‘one‐to‐two’ TCS working model for the role of ColS_XOCgx_4036_, ColR_XOCgx_4037_, and EmvR in modulating Xoc motility.

## Graphical abstract

An orphan single‐domain response regulator EmvR is co‐opted by a sensor kinase to form a branched two‐component system to modulate pili‐dependent motility.

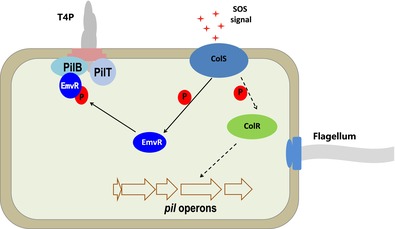

## 1 Introduction

The bacterial genus *Xanthomonas* comprises a broad range of gram‐negative plant pathogens that infect about 400 plant hosts, including some economically important crops (Niño‐Liu et al. 2006; An et al. 2020). One of the important members, *Xanthomonas oryzae* pv. *oryzicola* (Xoc), is the causal agent of bacterial leaf streak (BLS) of rice. Under conditions favourable for infection, BLS may cause rice yield reduction by 20%–32% (Niño‐Liu et al. 2006). Because BLS resistance in rice is a quantitatively inherited trait that is driven by multiple genes and the resistant rice cultivars (varieties) are rare or unstable (Ma et al. 2021; Fang et al. 2023), BLS is becoming increasingly severe and emerging as a devastating disease in certain rice‐producing areas such as South China and Southeast Asia.

Xoc serves as a model organism for understanding bacterial nonvascular plant pathogenesis (Wang et al. 2007). It enters rice leaves mainly via stomata or damaged parts of leaves. After infection, the Xoc cells multiply in the substomatal cavity and then colonise the intercellular spaces (apoplast) of the mesophyll, causing water‐soaked interveinal lesions that develop into yellowish‐grey and translucent streaks (Niño‐Liu et al. 2006). Like other phytopathogenic xanthomonads, this pathogen possesses multiple virulence‐related factors or systems, such as extracellular polysaccharide (EPS), lipopolysaccharide (LPS), extracellular enzymes (proteases, cellulases), cell motility, a quorum‐sensing system, the type III secretion system (T3SS) and so on (Niño‐Liu et al. 2006; Wang et al. 2007; An et al. 2020).

Type IV pili (T4P) are employed by a wide range of bacteria for translocation over moist surfaces. Structures, biogenesis and functions of T4P have been extensively studied in several human disease‐causing pathogens, including *Pseudomonas aeruginosa* , *Neisseria gonorrhoeae* , *Myxococcus xanthus* and *Escherichia coli* (Craig et al. 2019; Ellison et al. 2022). Besides motility across surfaces, T4P have been shown to participate in several physiological processes, such as virulence, biofilm formation, surface sensing and protein secretion. T4P are dynamic filaments that consist of a hair‐like polymer of thousands of subunits of a major pilin protein PilA and fewer minor pilins, and can be rapidly extended and retracted. Through repeated extension, tethering (attachment) and retraction of T4P, bacteria can move on a surface to which they are attached (Ellison et al. 2022). An ATPase called PilB provides the energy for pilus polymerisation and extension, while its paralogues PilT/PilU power pilus disassembly and retraction (Chiang et al. 2008; Jain et al. 2017; Chlebek et al. 2019). Despite great progress in understanding T4P biogenesis and function, which are known to be subject to environmental stimuli and internal signals such as cyclic di‐GMP (Dunger et al. 2016; Jain et al. 2017), knowledge about the mechanisms by which bacteria sense their environment to coordinate T4P biogenesis, extension/retraction, or drive surface motility remains poor.

In *Xanthomonas* spp., mechanisms of T4P biogenesis and function are thought to differ from those of bacteria causing human disease (Dunger et al. 2016). The c‐di‐GMP receptor protein FimX is regarded as a key regulator of T4P biogenesis (Dunger et al. 2016; Jain et al. 2017). FimX directly interacts with the ATPase PilB, the motor for T4P, in several bacterial species such as *P. aeruginosa* (Jain et al. 2017). In *Xanthomonas* species, PilZ interacts with both FimX and PilB to form the FimX‐PilZ‐PilB complex, which enhances PilB activity and regulates T4P biogenesis (Dunger et al. 2016; Llontop et al. 2021). Recently, the PdeK‐PdeR two‐component signalling system (TCS) was demonstrated to physically interact with FimX to form a T4P assembly complex. This complex is supposed to connect environmental cues to the T4P assembly (Wei et al. 2021).

Although T4P are crucial virulence factors for many animal‐pathogenic bacteria, their pathogenic effects vary among different *Xanthomonas* spp. Several types of bacterial motility influenced by T4P have been observed in *Xanthomonas* species. Twitching motility, a pilus‐dependent form of bacterial translocation over moist surfaces, has been described in Xoc, *X. oryzae* pv. *oryzae* (Xoo) and *X. citri* subsp. *citri* (Xcci). Mutants lacking functional T4P display reduced twitching motility (Dunger et al. 2014; Yu et al. 2020; Wei et al. 2021; Kumar Verma et al. 2024). These pathogens are supposed to be able to attach to surfaces of the host plant cells using a polar flagellum and T4P and move across the surface using twitching motility. Another type of motility that has been observed to be influenced by T4P is spreading (or sliding) motility, a passive translocation over moist surfaces, which is driven by the expansive forces of a growing colony and is supposed to be indirectly inhibited by T4P and flagella (Harshey 2003; Murray and Kazmierczak 2008). T4P‐deficient mutants in Xoc, Xoo and Xcci present obviously increased spreading on semisolid agar surfaces (Dunger et al. 2014; Yang et al. 2015; Wei et al. 2021), while the mutants of T4P‐related genes in *X. campestris* pv. *campestris* (Xcc) display reduced spreading (for review see Dunger et al. 2016).

The TCS, which is composed of at least a histidine kinase (HK) sensor and a response regulator (RR), is a major mechanism widely adopted by bacteria to sense environmental changes and modulate subsequent adaptive responses (Stock et al. 2000; Gao et al. 2019). Generally, as the output components of signalling pathways, prototypical RRs are composed of an N‐terminal phosphoryl‐accepting receiver (REC) domain and a variable C‐terminal output domain that generates a wide variety of cellular responses to the corresponding environmental signals (Gao et al. 2019). However, some RRs, the so‐called single‐domain response regulators (SD‐RRs), contain only a stand‐alone REC domain. Knowledge about their cellular functions is poor. So far, several SD‐RRs have been demonstrated to control cell motility, such as *E. coli* CheY involved in swimming (flagellum‐dependent) motility (Sarkar et al. 2010), *P. aeruginosa* PilG and PilH involved in twitching motility (Bertrand et al. 2010; Kühn et al. 2023), and Xcc VemR and McvR involved in swimming motility (Li et al. 2020, 2022). Here, we show that an orphan SD‐RR, encoded by *XOCgx_1445* in Xoc GX01 strain and named EmvR (*E*PS‐, *m*otility‐ and *v*irulence‐related *R*egulator), plays a role in virulence, EPS production and twitching and spreading motilities but not swimming motility. We provide evidence demonstrating that EmvR interacts with the extension motor ATPase PilB and represses the ATPase activity of PilB. Additionally, we found that EmvR can also bind to a TCS sensor HK named ColS_XOCgx_4036_, which is supposed to integrate environmental stimuli to the T4P assembly and movement.

## 2 Results

### 2.1 EmvR Is Involved in Twitching and Spreading Motility, EPS Production and Virulence

An earlier work aimed at investigating the functions of RRs in Xoc GX01 found that an insertional mutant of EmvR (*XOCgx_1445*), which was constructed by the integration method using the suicide plasmid (pK18*mob*) and named NK3121, presented slight changes in spreading motility (Zhang et al. 2018). EmvR is an atypical orphan SD‐RR of 134 amino acids in length; it contains a REC domain but lacks a conventional output effector domain. Amino acid sequence pairwise alignments using Vector NTI showed that EmvR shares 93.3% identity (97.0% similarity) with its counterpart XC_1160 in Xcc, 25.8% identity (45.7% similarity) with the characterised *E. coli* SD‐RR CheY, 23.8% identity (45.4% similarity) with the Xcc McvR and 18.3% identity (32.3% similarity) with the Xcc VemR, implying potentially different roles of these SD‐RRs in cell motility in *Xanthomonas* spp.

To explore the detailed function of EmvR in Xoc, we constructed an in‐frame marker‐free deletion mutant of the *emvR* gene in the wild‐type strain GX01 by allelic homologous recombination employing the suicide plasmid pK18mob*sacB* (Table S1), which was designated as Δ*emvR* (Table S1). Simultaneously, a complemented strain, named CΔ*emvR* (Table S1), was constructed by introducing the recombinant plasmid pXC*emvR*, which was derived from the *emvR* coding sequence cloned into the vector pXUK, into the mutant Δ*emvR*.

**FIGURE 1:**
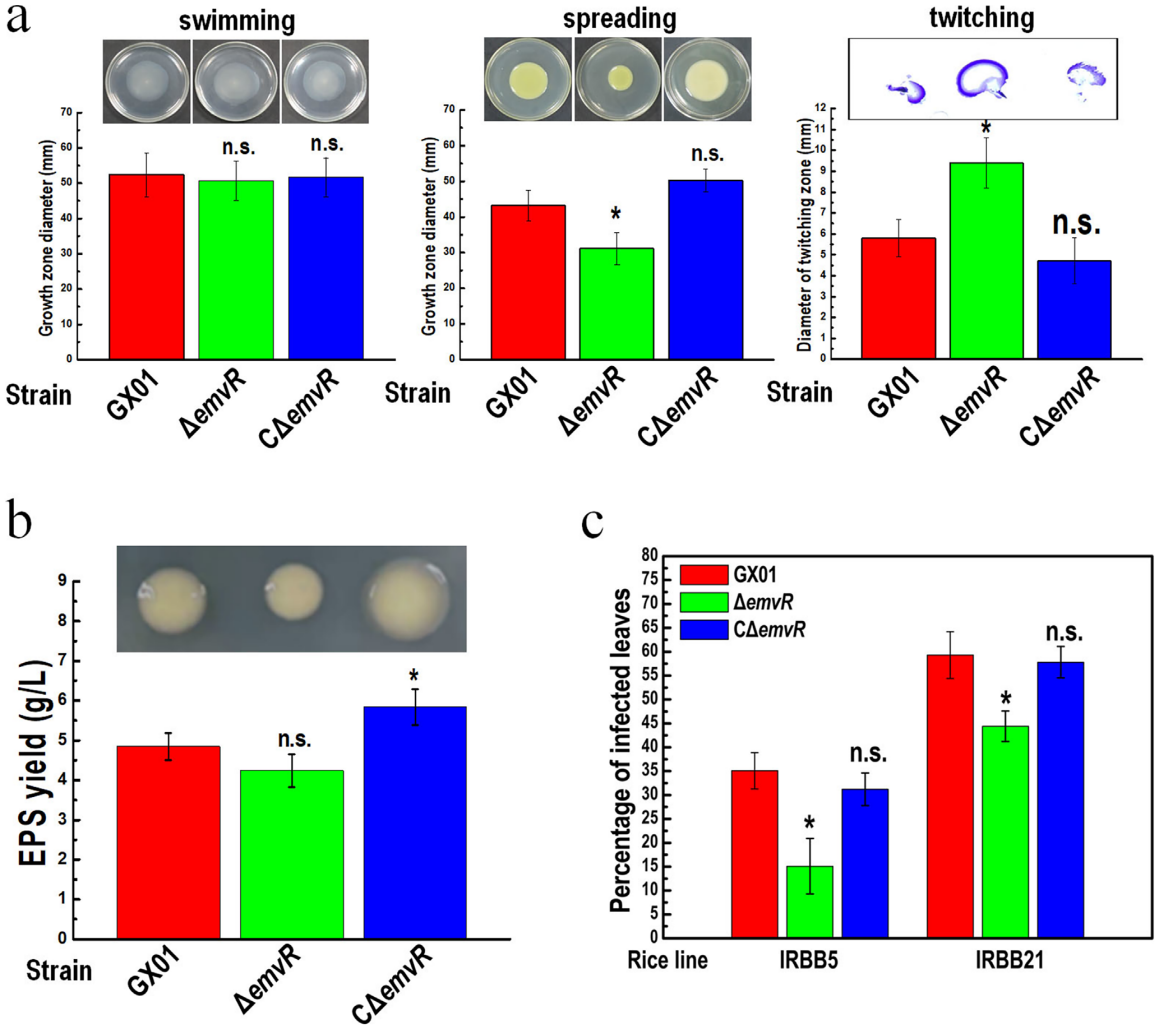
EmvR has impacts on cell motilities, extracellular polysaccharide (EPS) production and virulence in *Xanthomonas oryzae* pv. *oryzicola* (Xoc). (a) Mutation in *emvR* reduces spreading motility whereas it enhances twitching motility. Xoc strains were stabbed into ‘swimming’ plates, spotted onto ‘spreading’ plates, or punctured into NA plates to reach the plastic surface, followed by incubation for 4–5 days. For ‘twitching’ plates, the agar was removed, and the Petri plate was stained with crystal violet, then the unbound dye was rinsed thoroughly with water. The representative colony morphologies of Xoc strains were photographed and the colony diameters of each strain on the different media were measured. GX01, wild type; Δ*emvR*, deletion of *emvR*; CΔ*emvR*, complemented strain. Data shown are the mean and standard deviation (SD) from 15 measurements in a representative experiment. Significance was determined by analysis of variance (ANOVA) and Dunnett's post hoc test for comparison to the wild type. *p < 0.05; n.s., not significant. Similar results were obtained in two other independent experiments. (b) EmvR is involved in EPS production. Xoc strains were spotted onto ‘EPS’ plates and grown for 5 days, the representative colony morphologies of Xoc strains were photographed. EPS yield was measured using the method as previously described (Tang et al. 1991). Values given are the means ± SD of triplicate measurements from a representative experiment, significance was determined by ANOVA and Dunnett's post hoc test for comparison to the wild type. *p < 0.05; n.s., not significant. Similar results were obtained in two other independent experiments. (c) EmvR is involved in the infection in Xoc. Six‐week‐old rice lines IRBB5 and IRBB21 were used to assess the virulence of Xoc strains. Three replicates of each independent experiment were carried out. Ten days after inoculation the relative virulence was determined. Values given are the means ± SD of three replicates. Significance was determined by ANOVA and Dunnett's post hoc test for comparison to the wild type. *p < 0.05; n.s., not significant. Similar results were obtained in two other independent experiments.

To estimate the influences of *emvR* on cell motilities of Xoc, the above constructed strains were tested by inoculation on swimming plates (0.28% wt/vol agar) and spreading plates (0.6% wt/vol agar). The results showed that the *emvR‐*mutant strain Δ*emvR* had diminished spreading capacity with colony diameters about 28% smaller compared with the wild‐type strain, although the mutant had similar swimming capacity as the wild‐type (Figure 1a). The spreading motility of Δ*emvR* could be restored by complementation. As shown in Figure 1a, the complemented strain CΔ*emvR* even had enhanced spreading motility with colony diameters about 16% larger as compared with the wild‐type strain. Twitching motility, which is dependent on T4P and independent of flagella, between the agar and Petri dish interface of Xoc strains was also tested. The diameter of Δ*emvR* colonies on subsurface was increased by 62.1% as compared with the wild‐type strain, and the twitching motility of the Δ*emvR* mutant could be reduced back to the wild‐type level by the expression of the innate *emvR* gene carried on an ectopic plasmid (Figure 1a), suggesting that disruption of *emvR* in Xoc probably caused an increase in T4P movement.

In addition, we examined other phenotypes of the mutant Δ*emvR*, such as the production of EPS and the activities of the extracellular enzymes (protease, cellulase and amylase), which collectively contribute to virulence in *Xanthomonas* spp. during the disease process. The results showed that Δ*emvR* produced slightly less EPS compared with the wild type (Figure 1b). Notably, the EPS yield of the complemented strain CΔ*emvR* was significantly higher than that of the wild‐type strain (Figure 1b), indicating that EmvR most likely plays a positive regulatory role in the EPS production in Xoc. However, no difference was seen between Δ*emvR* and the wild type in the activities of extracellular enzymes (Figure S1).

To estimate whether deletion of *emvR* affects the pathogenesis of Xoc, the virulence of Δ*emvR* was tested in the rice varieties IRBB5 and IRBB21 by the infiltration method. The results showed that Δ*emvR* caused a similar lesion length to the wild type in the tested rice varieties (data not shown), indicating that deletion of *emvR* did not affect the virulence of Xoc when the bacterial cells were introduced into rice tissues by infiltration. However, when the bacterial cells were introduced into the rice plants by spraying, a difference in virulence between the mutant strain Δ*emvR* and the wild‐type strain was seen in the tested rice plants 10 days after inoculation. The percentage of diseased leaves in both rice varieties IRBB5 and IRBB21 in response to Δ*emvR* infection was significantly lower compared with the infection by the wild type (Figure 1c). These results revealed that deletion of *emvR* in Xoc caused a significant reduction in virulence to the rice plants of IRBB5 and IRBB21 inoculated by leaf spraying but not leaf infiltration, suggesting that the Xoc EmvR plays a role in the early invasion phase of the disease cycle.

### 2.2 Overexpression of *emvR* in Xoc Enhances EPS Production and Spreading Motility but Reduces Twitching Motility

The above data demonstrated that the complementation of Δ*emvR* mutant with plasmid‐borne full‐length *emvR* gene enhanced EPS yield to a level exceeding that of the wild type (Figure 1b), implying that EmvR most likely controls EPS production in Xoc. To gain more knowledge about the function of the EmvR protein, we further tested the effects caused by overexpression of *emvR*.

**FIGURE 2:**
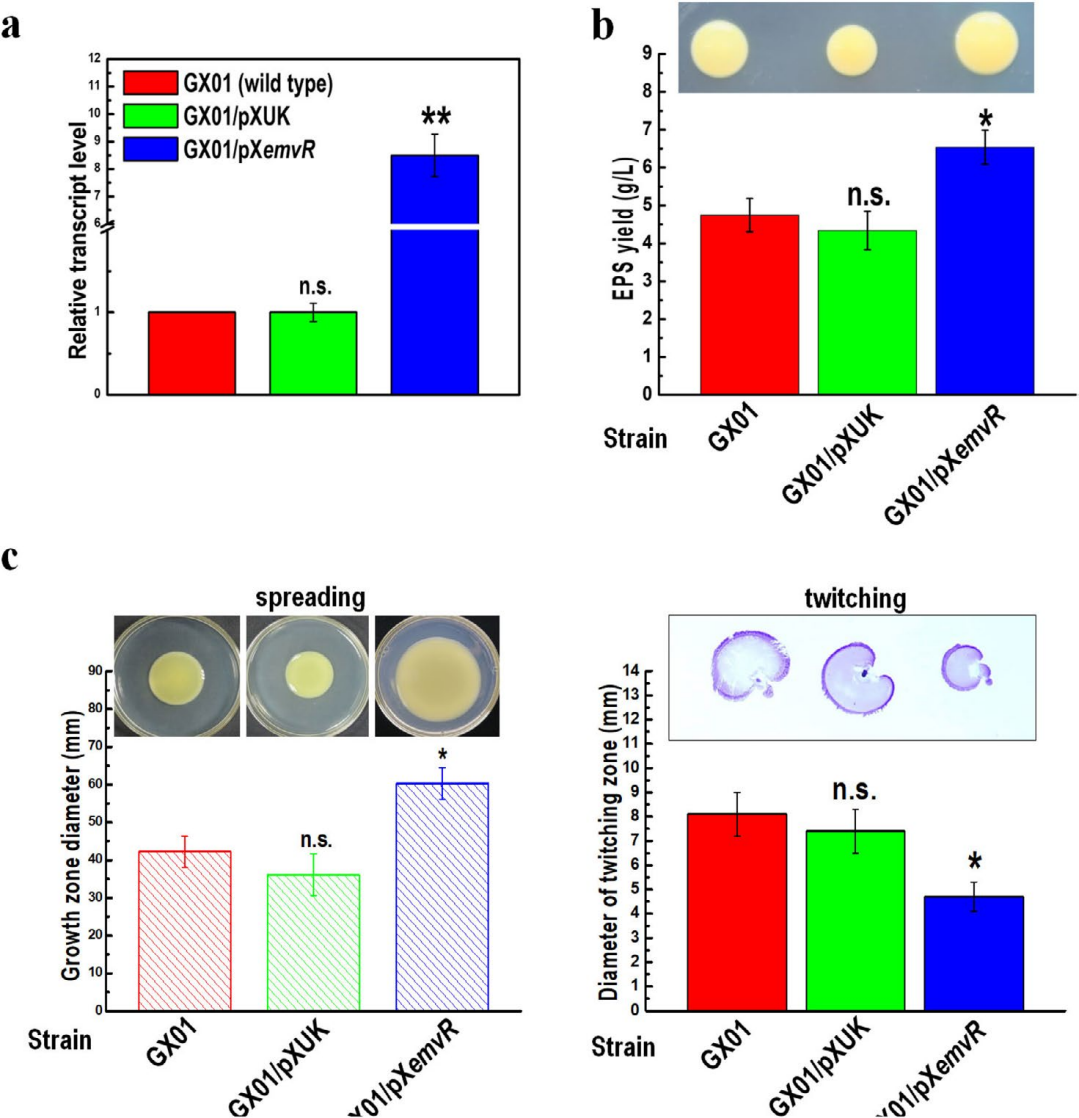
Overexpression of *emvR* alters extracellular polysaccharide (EPS) production and spreading and twitching motilities in *Xanthomonas oryzae* pv. *oryzicola* (Xoc). (a) Examination of the expression level of *emvR* in Xoc strains. Reverse transcription‐quantitative PCR (RT‐qPCR) assay to measure the *emvR* transcript levels. Xoc strains GX01, GX01/pXUK, and GX01/pX*emvR* were cultured in NB medium to an optical density at 600 nm (OD_600_) of 1.0, and RNAs were extracted. The RT‐qPCR tests were performed in triplicate and the relative mRNA level was calculated. Values given are the mean ± SD from triplicate measurements in a representative experiment. **p < 0.01; n.s., not significant. (b) EPS production of different Xoc strains. Xoc strains grown on ‘EPS’ plates for 5 days; the representative colony morphologies of Xoc strains were photographed. EPS yield was further quantitatively measured. Values given are the means ± SD of triplicate measurements from a representative experiment; significance was determined by analysis of variance (ANOVA) and Dunnett's post hoc test for comparison to the wild type. *p < 0.05; n.s., not significant. Similar results were obtained in two other independent experiments. (c) Cell motility of different Xoc strains. Cultures of each Xoc strain were inoculated on ‘spreading’ and into ‘twitching’ medium, and cultured for 4 and 7 days. The representative colony morphologies of Xoc strains were photographed and colony diameters of each strain on the different media were measured. Values given are the mean ± SD of 15 measurements from a representative experiment. Significance was determined by ANOVA and Dunnett's post hoc test for comparison to the wild type. *p < 0.05; n.s., not significant. The experiment was repeated three times with similar results.

To do this, the recombinant plasmid pXC*emvR* (Table S1) was introduced into the Xoc wild‐type strain GX01, resulting in strain GX01/pXC*emvR* (Table S1). Simultaneously, the empty vector pXUK (Table S1) was introduced into the wild‐type strain GX01 to generate the control strain GX01/pXUK (Table S1). The expression levels of *emvR* in Xoc strains GX01, GX01/pXUK, and GX01/pXC*emvR* were assessed by reverse transcription‐quantitative PCR (RT‐qPCR). The transcription level of *emvR* in GX01/pXC*emvR* was approximately eightfold higher compared with the control strain GX01/pXUK, which expressed a similar *emvR* transcription level as the wild‐type strain GX01 (Figure 2a).

A series of phenotypes of the overexpressing strain GX01/pXC*emvR* were tested. Results revealed that GX01/pXC*emvR* produced more EPS (Figure 2b) and exhibited increased spreading motility and weakened twitching motility (Figure 2c) compared with the wild type. However, no obvious difference was seen among the tested strains in swimming motility and extracellular enzyme activity (Figure S2). Taken together, these data demonstrate that overexpression of *emvR* in Xoc has a positive effect on EPS production and spreading motility but a negative effect on twitching motility.

### 2.3 EmvR Interacts With the Pili Motor Protein PilB and the TCS Sensor HK ColS_XOCgx_4036_

Based on the bioinformatics data that EmvR belongs to the SD‐RRs group that are supposed to carry out their regulatory functions via interacting with target proteins, we investigated whether EmvR interacts with other proteins in Xoc cells. To this purpose, co‐immunoprecipitation (co‐IP) coupled with liquid chromatography tandem‐mass spectrometry (LC–MS/MS) was carried out as previously described (Li, Peng, et al. 2023) to search for the potential target proteins of EmvR. Xoc GX01 chromosomally expressing EmvR fused with a 3 × FLAG‐tag (EmvR:3 × FLAG) was first generated and named GX01(EmvR:3 × FLAG) (Table S1). A western blot assay revealed that the EmvR:3 × FLAG fusion protein could be eluted from the reporter strain GX01(EmvR:3 × FLAG) but not from the wild‐type strain GX01 (Figure S1). Protein complexes with EmvR:3 × FLAG in the cells of GX01(EmvR:3 × FLAG) strain were purified and analysed by LC–MS/MS. Two experiments of co‐IP coupled with LC–MS/MS were performed, and a number of proteins identified in both experiments were taken as potential targets of EmvR (Table S2) for further studies. These proteins include seven chemotaxis‐related proteins (XOCgx_2212, XOCgx_2478, XOCgx_2601, XOCgx_2603, XOCgx_2604, XOCgx_2606 and XOCgx_2861), three pilus‐related proteins (XOCgx_1113/PilO, XOCgx_1260/PilB and XOCgx_3448/PilU), three TCS members (XOCgx_0533, XOCgx_1563 and XOCgx_4036/ColS_XOCgx_4036_), and two transcriptional regulators or DNA‐binding proteins (XOCgx_2510 and XOCgx_3167).

**FIGURE 3:**
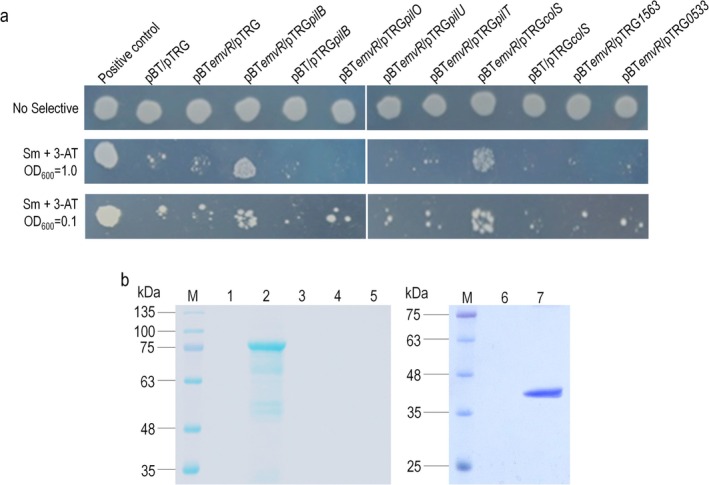
Confirmation of EmvR physical interactions. (a) Bacterial two‐hybrid experiment. The reporter strains *Escherichia coli* XL1‐Blue MRF′ with different plasmid pairs were grown on nonselective plates (inoculated with a cell concentration of OD_600_ of 1.0) and double‐selection indicator plates (inoculated with cell concentrations of OD_600_ of 1.0 and 0.1) containing 3‐amino‐1,2,4‐triazole (3‐AT) and streptomycin (Sm). The reporter strain with the plasmid pair pBT*emvR*‐pTRG*pilB*, or pBT*emvR*‐pTRG*colS* could grow on double‐selective indicator plates. Three independent experiments showed similar results. (b) Pull‐down assay. 6 × His‐tagged EmvR protein and other 6 × His‐tagged proteins were overexpressed and purified. Bait protein EmvR was biotinylated and immobilised to streptavidin Sepharose beads. The full‐length or truncated potential prey proteins such as PilB, PilT, and ColS_XOCgx_4036_ were mixed with the bait protein and incubated. After elution, samples were separated on 12% SDS‐PAGE and visualised by Coomassie blue staining. Lane 1, 6 × His::PilB was mixed with streptavidin Sepharose beads (negative control); lane 2, pull‐down of 6 × His:PilB by biotinylated EmvR; lane 3, biotinylated EmvR was mixed with 6 × His:PilO; lane 4, biotinylated EmvR was mixed with 6 × His:PilU; lane 5, biotinylated EmvR was mixed with 6 × His:PilT; lane 6, 6 × His:ColS_XOCgx_4036_ was mixed with streptavidin Sepharose beads (negative control); lane 7, pull‐down of 6 × His:ColS_XOCgx_4036_ by biotinylated EmvR; M, molecular mass marker. Three independent experiments showed the same result.

Given that EmvR most likely affects T4P‐dependent motility, we first validated its interaction with the motility‐related proteins using a bacterial two‐hybrid (B2H) assay as previously described (Li et al. 2020). The genes encoding the identified three pilus‐related proteins (*pilO*, *pilB* and *pilU*) and the *emvR* gene were cloned into the prey vector pTRG and the bait vector pBT, respectively, resulting in the recombinant bait plasmid pBT*emvR* and recombinant prey plasmids pTRG*pilO*, pTRG*pilB* and pTRG*pilU* (Table S1). The reporter *E. coli* XL1‐Blue MRF′ strain (Table S1) was co‐transformed with the bait plasmid (pBT or its derivative) and the prey plasmid (pTRG or its derivatives) and tested on double‐selective indicator plates. The *E. coli* strain co‐expressing EmvR and PilB could grow on the selective plates (Figure 3a), indicating that EmvR interacts with PilB under the test conditions. In contrast, no interaction was observed between EmvR and PilO or PilU. Recombinant 6 × His‐tagged proteins were also overproduced and purified, and pull‐down biotinylated protein–protein assays were carried out to examine the EmvR interaction in vitro. As shown in Figure 3b, the 6 × His‐tagged EmvR could capture PilB but not PilO or PilU, supporting a physical interaction of EmvR with PilB. It is known that in most bacteria PilB is an ATPase and responsible for the assembly/extension of the pilus, while its paralogue PilT is involved in pilus disassembly/retraction (Chlebek et al. 2019). Whether EmvR interacts with PilT was further investigated by using both B2H and pull‐down assays. However, no interaction was observed under the test conditions (Figure 3).

As a TCS RR, EmvR was believed to mediate intracellular or extracellular signals received by a sensor protein. Therefore, we further validated the interaction of EmvR with the above‐identified TCS proteins ColS_XOCgx_4036_, XOCgx_1563 and XOCgx_0533 by B2H assay. EmvR interacted with the sensor HK ColS_XOCgx_4036_, but not the proteins XOCgx_1563 and XOCgx_0533 under the test conditions (Figure 3). Pull‐down assay further substantiated that EmvR interacted with ColS_XOCgx_4036_ in vitro (Figure 3b). Possible interaction of EmvR with the above‐identified seven chemotaxis proteins annotated as methyl‐accepting chemotaxis proteins (MCPs) was also tested by B2H assay. However, the result revealed that EmvR did not interact with any of these proteins (Figure S2).

Taken together, the above data demonstrate that EmvR interacts with the sensor HK ColS_XOCgx_4036_ and the pilus motor protein PilB, suggesting that these proteins may comprise a regulatory pathway.

### 2.4 EmvR Functions Downstream of ColS_XOCgx_4036_ in Controlling Cell Motility

The TCS ColS/ColR has been demonstrated to play vital roles in several pathogenic bacteria. A scan of the genome sequence of Xoc GX01 revealed that there are three ColS/ColR TCSs: ColS_XOCgx_1237_/ColR_XOCgx_1236_, ColS_XOCgx_3645_/ColR_XOCgx_3646_ and ColS_XOCgx_4036_/ColR_XOCgx_4037_. To investigate the functions of ColS_XOCgx_4036_/ColR_XOCgx_4037_ in Xoc, mutant strains with deletion of the *colS*_*XOCgx_4036*_ and *colR*_*XOCgx_4037*_ genes in the wild‐type strain GX01 were constructed and designated as Δ*colS*_*XOCgx_4036*_ and Δ*colR*_*XOCgx_4037*_, respectively (Table S1). Simultaneously, their complemented strains named CΔ*colS*_*XOCgx_4036*_ and CΔ*colR*_*XOCgx_4037*_ were also constructed by introducing a recombinant plasmid (pXC*colS*_*XOCgx_4036*_ or pXC*colR*_*XOCgx_4037*_), derived from cloning the corresponding gene into the vector pXUK, into the mutant strains Δ*colS*_*XOCgx_4036*_ and Δ*colR*_*XOCgx_4037*_, respectively (Table S1).

**FIGURE 4:**
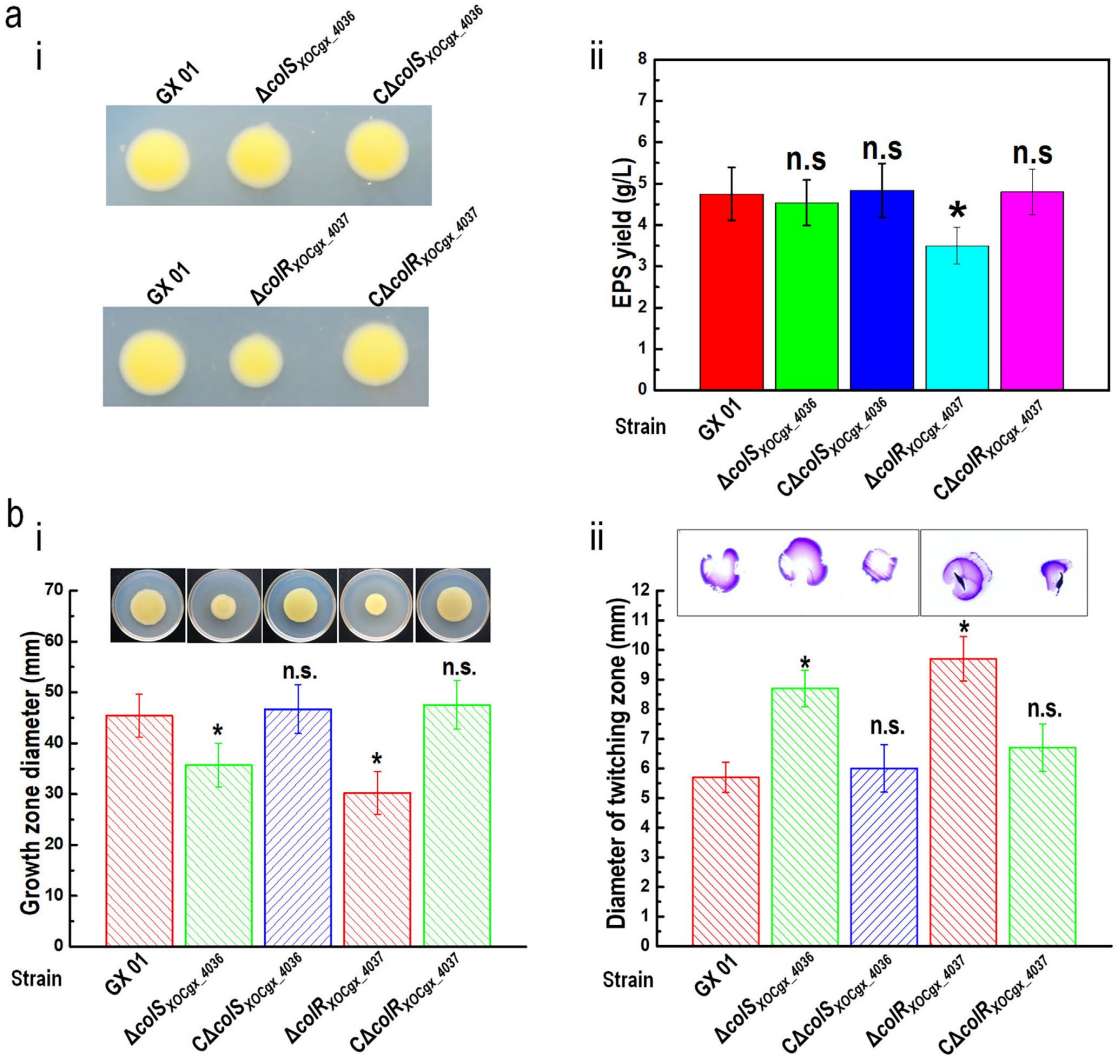
ColS_XOCgx_4036_/ColR_XOCgx_4037_ system controls cell motility in *Xanthomonas oryzae* pv. *oryzicola* (Xoc). (a) Mutation in *colR*_*XOCgx_4037*_ reduces extracellular polysaccharide (EPS) production. (i) Xoc strains were spotted onto ‘EPS’ plates and grown for 5 days. The representative colony morphologies of Xoc strains were photographed. (ii) Xoc strains cultured in medium for 3 days, and EPS was precipitated from the culture supernatant. Values given are the means ± SD of triplicate measurements from a representative experiment; significance was determined by analysis of variance (ANOVA) and Dunnett's post hoc test for comparison to the wild type (GX01). *p < 0.05; n.s., not significant. Similar results were obtained in two other independent. (b) Mutation in *colS*_*XOCgx_4036*_ or *colR*_*XOCgx_4037*_ alters cell motilities. Xoc strains were inoculated onto ‘spreading’ plates (i) and into ‘twitching’ plates (ii), respectively, followed by incubation for 5 and 7 days. The representative colony morphologies of Xoc strains were photographed and the colony diameters of each strain on the different media were measured. Data shown are the means ± SD from 15 measurements in a representative experiment. Significance was determined by ANOVA and Dunnett's post hoc test for comparison to the wild type. *p < 0.05; n.s., not significant. Similar results were obtained in two other independent experiments.

A series of tests was conducted to assess the influence of mutation in *colR*_*XOCgx_4037*_ or *colS*_*XOCgx_4036*_ on EPS production and cell swimming, spreading and twitching motility. As shown in Figure 4, the RR deletion mutant strain Δ*colR*_*XOCgx_4037*_ produced 27.7% less EPS and had weakened spreading but enhanced twitching motility compared with the wild type. Moreover, the complemented strain CΔ*colR*_*XOCgx_4037*_ had similar EPS yield and motility phenotypes as the wild‐type strain (Figure 4), indicating that the phenotypes of the deletion mutant could be restored back to the wild type by the *colR*_*XOCgx_4037*_ gene expressed in trans. These data revealed that the TCS member ColR_XOCgx_4037_ regulates EPS production and cell motility in Xoc. Interestingly, the HK mutant strain Δ*colS*_*XOCgx_4036*_ produced a similar EPS yield but had an obvious change in motilities (weakened spreading and enhanced twitching) compared with the wild type (Figure 4), and the motilities of the complemented strain CΔ*colS*_*XOCgx_4036*_ were similar to the wild type under the test conditions, indicating that the HK ColS_XOCgx_4036_ modulates cell motility but not EPS production in Xoc. These data reveal that ColS_XOCgx_4036_ and ColR_XOCgx_4037_ regulate spreading and twitching motility in the same manner, although they have divergent effects on EPS production.

**FIGURE 5:**
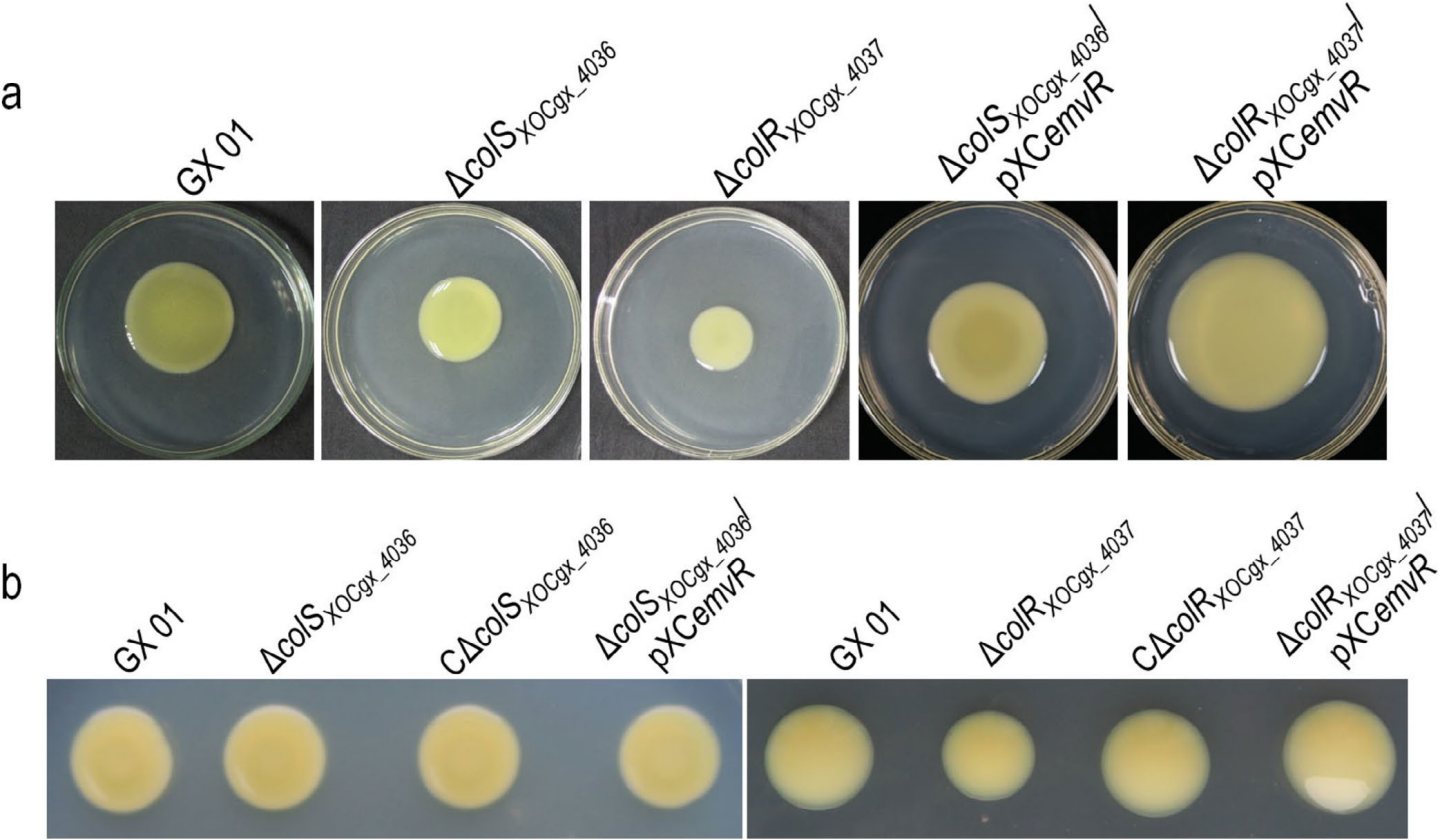
Overexpression of *emvR* in the *colS*_*XOCgx_4036*_ and *colR*_*XOCgx_4037*_ deletion mutants can restore their phenotypes to a level similar to and exceeding that of the wild type. *Xanthomonas oryzae* pv. *oryzicola* (Xoc) wild‐type strain GX01, *colS*_*XOCgx_4036*_ deletion mutant Δ*colS*_*XOCgx_4036*_, *colR*_*XOCgx_4037*_ deletion mutant Δ*colR*_*XOCgx_4037*_, and cross‐complemented strains Δ*colS*_*XOCgx_4036*_/pXC*emvR* and Δ*colR*_*XOCgx_4037*_/pXC*emvR* were spotted on ‘spreading’ plates (a) and NA plates containing 2% sucrose (b), and incubated for 5 days. The strain Δ*colR*_*XOCgx_4037*_/pXC*emvR* displayed larger colony sizes than that of the wild type on ‘spreading’ plates.

To investigate the influence of EmvR on the regulatory effects of ColS_XOCgx_4036_/ColR_XOCgx_4037_ in Xoc, we employed an expression experiment where *emvR* was overexpressed in the mutant strains ∆*colS*_*XOCgx_4036*_ and ∆*colR*_*XOCgx_4037*_. The plasmid pXC*emvR* was introduced into ∆*colS*_*XOCgx_4036*_ and ∆*colR*_*XOCgx_4037*_ to generate the recombinant strains named ∆*colS*_*XOCgx_4036*_/pXC*emvR* and ∆*colR*_*XOCgx_4037*_/pXC*emvR* (Table S1), respectively. The spreading motility and EPS production of these recombinant strains were tested. As shown in Figure 5, ∆*colR*_*XOCgx_4037*_/pXC*emvR* produced the wild‐type level EPS yield and had obviously stronger spreading motility than the wild‐type strain. However, the EPS production and spreading motility of ∆*colS*_*XOCgx_4036*_/pXC*emvR* were similar to that of the wild type (Figure 5). These data implied that additionally expressing EmvR in ∆*colS*_*XOCgx_4036*_ and ∆*colR*_*XOCgx_4037*_ could restore EPS production and spreading motility. The phenotypic effects of additionally expressing *colS*_*XOCgx_4036*_ or *colR*_*XOCgx_4037*_ in the deletion mutant Δ*emvR* was also tested. Results showed that the recombinant strains ∆*emvR*/pXC*colS*_*XOCgx_4036*_ and ∆*emvR*/pXC*colR*_*XOCgx_4037*_ had similar EPS production and spreading motility as Δ*emvR* (Figure S3), indicating that additionally expressing *colS*_*XOCgx_4036*_ or *colR*_*XOCgx_4037*_ in Δ*emvR* could not restore EPS production and spreading motility.

Taken together, the above combined data suggest that EmvR may function downstream of ColS_XOCgx_4036_ in the signalling pathway that controls Xoc cell motility, and EmvR regulatory activity is most likely to be modulated by the sensor HK ColS_XOCgx_4036_.

### 2.5 EmvR Represses the Activity of the Pilus Motor Protein PilB

**FIGURE 6:**
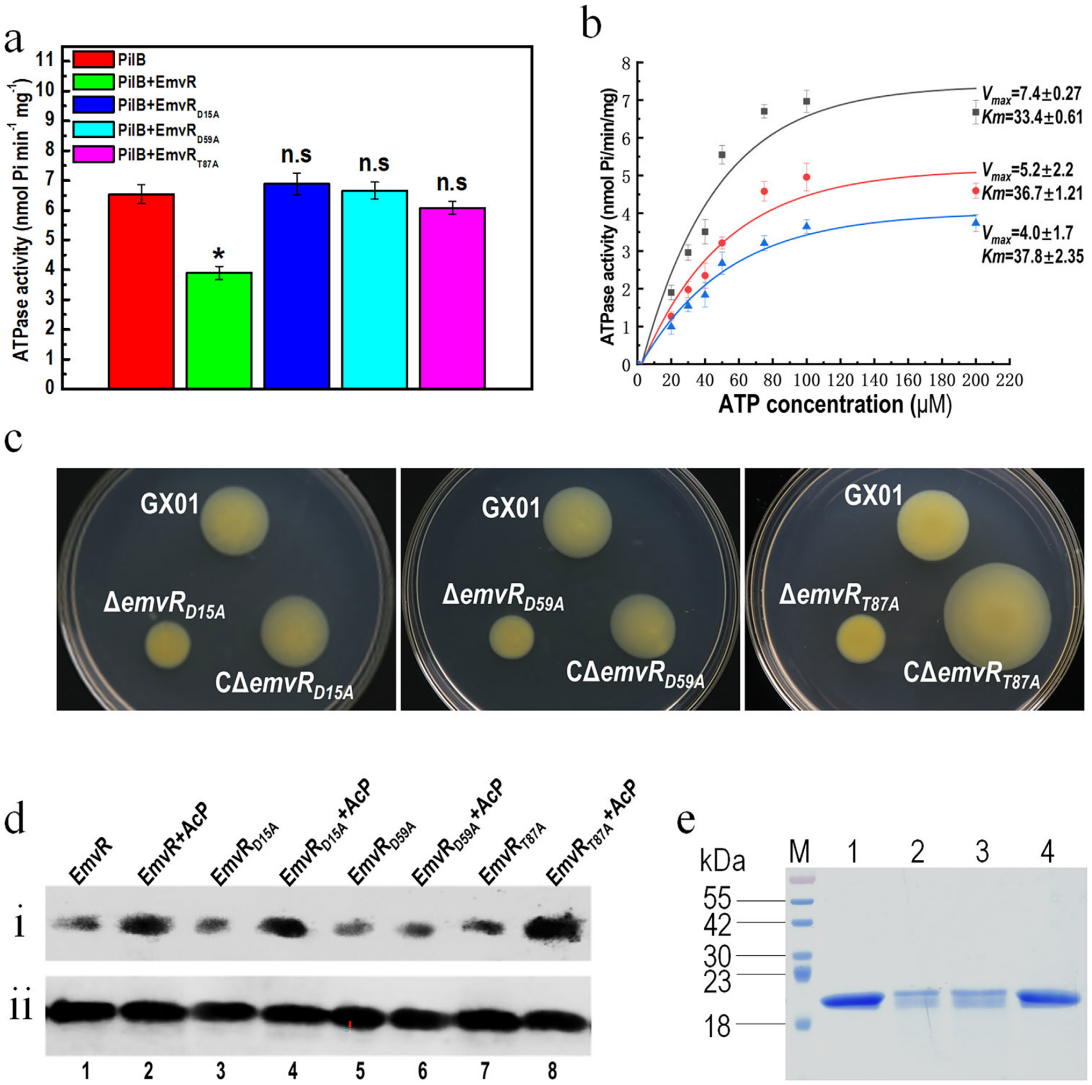
EmvR affects the ATPase activity of PilB, and substitution of Asp‐15, Asp‐59 or Thr‐87 in EmvR causes a loss of its regulatory functions. (a) The ATPase activity of PilB in the presence of wild‐type EmvR or EmvR variants. Two micrograms of 6 × His:PilB protein was incubated with 1 mM ATP for 30 min, the value of A_630_ was measured and converted into inorganic phosphate (P_i_) released. When 2 μg of wild‐type EmvR protein was added into the reaction mix, the ATPase activity of PilB was decreased. When wild‐type EmvR was substituted with its variants, no significant change of ATPase activity was observed. Values given are the means ± SD of triplicate measurements from a representative experiment, significance was determined by analysis of variance (ANOVA) and Dunnett's post hoc test for comparison to the wild‐type. *p < 0.05; n.s., not significant. Similar results were obtained in two other independent. (b) Michaelis–Menten plots. ATPase activities of PilB with various concentrations of substrate ATP and inhibitor EmvR were measured. Each data point represents the means and SD from triplicate measurements. Each plot describes different EmvR concentrations 0 nM (black ■), 240 nM (red ●) and 480 nM (blue ▲). (c) Motility test of *Xanthomonas oryzae* pv. *oryzicola* (Xoc) strains. Xoc wild‐type strain (GX01), point mutant strains and complemented strains were inoculated on the ‘spreading’ plates and grown for 4 days. The representative colonies were photographed. (d) In vitro phosphorylation assays of EmvR and its variants. (i) Three micrograms of purified wild‐type EmvR and its variants produced in *Escherichia coli* were transferred to a PVDF membrane and probed for phosphorylation using Phos‐tag BTL‐104‐bound HRP‐SA. A clear phosphorylation signal was seen for non‐treated proteins (lane 1, 3, 5, 7). When incubated with acetyl‐phosphate (AcP) (lane 2, 4, 6, 8), no enhanced density band could be seen for the mutant EmvR_D59A_. (ii) Western blotting probed with antibody against the His‐tag showed that equal amounts of proteins were present in all samples. (e) Pull‐down assays of PilB with EmvR and its variants. Bait protein 6 × His:PilB was biotinylated and immobilised to streptavidin Sepharose beads. EmvR and its variants were mixed with the bait protein and incubated. After elution, samples were separated and visualised. Lane 1, pull‐down of EmvR by 6 × His:PilB; lane 2, pull‐down of EmvR_D15A_ by 6 × His:PilB; lane 3, pull‐down of EmvR_D59A_ by 6 × His:PilB; lane 4, pull‐down of EmvR_T87A_ by 6 × His:PilB; M, molecular mass marker. Three independent experiments showed the same result.

PilB homologues in several bacterial species have been demonstrated to possess ATPase activity (Jakovljevic et al. 2008; Llontop et al. 2021). To examine whether the PilB of Xoc has ATPase activity in vitro, the ATPase activity of the purified 6 × His‐tagged PilB was tested using a coupled enzyme assay that detects the release of phosphate from ATP. As shown in Figure 6a, phosphate release was observed when the 6 × His‐tagged PilB protein was incubated with ATP, demonstrating that Xoc PilB has ATPase activity in vitro. The ATPase activity of PilB was ~ 6.54 ± 0.32 nmol phosphate/min per mg PilB protein (nmol Pi min^−1^ mg^−1^) under the test conditions. The influence of EmvR on the activity of PilB was further examined. Upon addition of EmvR, an ~40.4% decrease in ATPase activity was observed (3.90 ± 0.22 nmol Pi min^−1^ mg^−1^) (Figure 6a), indicating that EmvR has a negative effect on PilB activity. To determine the mechanism of the inhibition of EmvR against PilB, the ATPase activities of PilB alone and mixed with 240 and 480 nM EmvR were measured with a range of ATP concentrations. The Michaelis–Menten kinetic parameters were then evaluated. As shown in Figure 6b, the *V*_max_ values were reduced in the presence of EmvR in a concentration‐dependent manner with almost no change in the *K*_m_ value, implying that EmvR is a non‐competitive inhibitor. These data further support the view that SD‐RRs function by directly interacting with their target proteins and allosterically modulating the targets' activity (Gao et al. 2019).

To evaluate the influence of PilB activity on cell motility in Xoc, a deletion mutant of the *pilB* gene was constructed from the wild‐type strain GX01 and designated as Δ*pilB* (Table S1). Simultaneously, a complemented strain named CΔ*pilB* was also constructed by introducing a recombinant plasmid carrying the *pilB*‐coding sequence into Δ*pilB*. Twitching and spreading motilities of the constructed strains were tested. As shown in Figure S4, Δ*pilB* displayed a severe reduction (almost a complete loss) in twitching motility and a great increase in spreading motility, compared with the wild type. Furthermore, the motilities of Δ*pilB* were restored to the wild‐type level by complementation (Figure S4).

Overall, the above combined data suggest that Xoc EmvR regulates twitching and spreading motilities via binding to PilB and repressing its activity.

### 2.6 Several Conserved Amino Acid Residues Are Essential for EmvR Regulatory Function, and the Aspartate 59 Is the Phosphorylation Site of EmvR

According to the previous studies on RR activation (for a review, see Gao et al. 2019), we conjectured that the aspartyl residues at position 15 (Asp‐15) and 59 (Asp‐59), and the threonine residue at position 87 (Thr‐87) might be vital for the function of EmvR. Asp‐15 most likely plays a key role in coordinating the catalytic magnesium ion (Mg^2+^ cation) at the reaction site, Asp‐59 may be the phosphorylation site, and Thr‐87 probably functions in the interaction with target proteins. To test these assumptions, mutant strains of *emvR* with its residues at positions 15, 59 and 87 replaced with alanine were created. The resulting Xoc derivatives named Δ*emvR*_*D15A*_, Δ*emvR*_*D59A*_ and Δ*emvR*_*T87A*_ were first examined for the expression level of the point‐mutated *emvR* gene using RT‐qPCR. The point mutations in the *emvR* gene had no obvious impact on its own transcription (Figure S7). Cell spreading motility was then tested. As shown in Figure 6b, all the three point mutation derivatives displayed reduced spreading motility, and the cell motility of their complemented mutant strains was restored to the wild‐type level, or even to a level exceeding the wild type. These data indicated that a single amino acid substitution at Asp‐15, Asp‐59 or Thr‐87 in EmvR could have a great impact on its regulatory function.

To further investigate the role of the three residues in EmvR protein, the *emvR* sequences with the above point mutations (*emvR*_D15A_, *emvR*_D59A_ and *emvR*_T87A_) were cloned into the expression vector pET‐30a. Wild‐type EmvR and the point mutation variants EmvR_D15A_, EmvR_D59A_ and EmvR_T87A_ were overproduced and purified. Purified proteins were run on an SDS‐PAGE and probed for phosphorylation using Phos‐tag‐BTL‐104. As seen in Figure 6c (lanes 1, 3, 5 and 7), a clear signal for phosphorylation is evident for EmvR and its variants EmvR_D15A_, EmvR_D59A_ and EmvR_T87A_, implying that EmvR and its variants are most likely phosphorylated by an unknown mechanism at a basal level in *E. coli* cells. When these 6 × His‐tagged proteins were incubated with acetyl‐phosphate (AcP), which specifically phosphorylates the acceptor aspartyl residues of many RRs (Wolfe 2010), more dense bands were observed for the wild‐type EmvR, as well as its variants EmvR_D15A_ and EmvR_T87A_ (lanes 2, 4 and 8), indicating EmvR and its variants EmvR_D15A_ and EmvR_T87A_ could be phosphorylated by AcP. However, no enhanced phosphorylation level of the variant EmvR_D59A_ was observed (lane 6), indicating that Asp‐59 is essential for EmvR phosphorylation.

Influences of the point mutation variant proteins on the activity of PilB were tested. As shown in Figure 6a, in contrast to the innate EmvR, when the EmvR_D15A_, EmvR_D59A_ or EmvR_T87A_ was added to the reaction mixture, no obvious decrease of PilB activity was observed, indicating that Asp‐15, Asp‐59 or Thr‐87 was essential for the biochemical action of EmvR; substitutions at Asp‐15, Asp‐59 or Thr‐87 in EmvR abolished its ability to modulate the targets' activity. Additionally, interactions of these variants with PilB were also examined using pull‐down assays. Interestingly, as seen in Figure 6e, under the test conditions, the amount of EmvR_T87A_ captured by PilB was similar to that of the wild‐type EmvR, while the amount of EmvR_D15A_ and EmvR_D59A_ captured was less than that of the wild‐type EmvR, indicating that replacing Asp‐15 or Asp‐59, but not Thr‐87, in EmvR reduces its binding ability to the target protein PilB.

Taken together, these combined data indicate that although how these point mutations alter the EmvR conformation and the mechanism by which EmvR modulates PilB activity remain unclear, Asp‐15, Asp‐59 or Thr‐87 in EmvR are essential for its biochemical and regulatory functions.

## 3 Discussion

In this study, we have demonstrated the involvement of the orphan SD‐RR EmvR in the virulence, EPS production and motility of Xoc. Interestingly, the *emvR*‐deleted mutant exhibited a significant reduction in the virulence of Xoc when the bacterial cells were sprayed onto rice leaves. As mentioned above, Xoc cells infect rice leaves through stomata and wounds. These data reveal that deletion of *emvR* may negatively affect Xoc invasion from stomata into leaf tissues but not the invasion by infiltration, suggesting that EmvR is important for the early stage of Xoc infection through stomata.

Several SD‐RRs have been demonstrated to control cell motility via regulating intermolecular effectors, such as *E. coli* CheY, which binds to the flagellar motor protein FliM/FliN to alter the flagellar rotation (Sarkar et al. 2010), *P. aeruginosa* PilG and PilH, which interact with T4P motor proteins PilB and PilT to control twitching motility (Bertrand et al. 2010; Kühn et al. 2023), and Xcc VemR, which binds to FliM to mediate swimming motility (Li et al. 2020). Here, we showed that deletion and overexpression of *emvR* in Xoc enhanced and weakened twitching motility, respectively (Figures 1 and 2). Moreover, our co‐IP, B2H and protein pull‐down assays demonstrated that EmvR physically interacts with PilB, a motor protein with ATPase activity. Further in vitro ATPase activity assays and point mutation analysis revealed that the binding of EmvR to PilB could significantly decrease the ATPase activity of PilB. It is known that PilB provides the energy for T4P extension and polymerisation (Chiang et al. 2008; Jain et al. 2017; Chlebek et al. 2019). As mentioned above, the T4P are responsible for twitching motility in *Xanthomonas* spp. (Dunger et al. 2014; Yu et al. 2020; Wei et al. 2021; Kumar Verma et al. 2024). Combined with these data, we suggest that EmvR plays a vital role in modulating Xoc T4P synthesis. Possibly, when necessary, EmvR binds to PilB to stop supplying the energy for T4P synthesis, thereby weakening the twitching motility of Xoc cells. In contrast to twitching, deletion and overexpression of *emvR* in Xoc weakened and enhanced cell spreading, respectively (Figures 1 and 2), implying that EmvR has a positive effect on spreading motility of Xoc. As described above, spreading motility is supposed to be indirectly inhibited by T4P and flagella (Harshey 2003; Murray and Kazmierczak 2008). This motility is also correlated with the production of surface‐wetting substances, such as lipopeptides, LPS and EPS. Given that mutation of EmvR slightly affects the EPS production, it is possible that the effect of EmvR on Xoc spreading is mainly achieved through regulating T4P biogenesis. Though T4P is a virulence factor in *Xanthomonas* species, the role of T4P in the interactions of bacteria–host varies in different *Xanthomonas* species (Dunger et al. 2016). The absence of a functional T4P in Xoo, the closely related pathovar of Xoc in rice, resulted in reductions in bacterial attachment, in planta migration, virulence in rice plants and even hypersensitive response induction in non‐host plants (Lim et al. 2008; Yang et al. 2015; Yu et al. 2020). Both Xoc and Xoo are thought to be able to attach to the surfaces of host cells using a polar flagellum and dynamic T4P, and to move across the surfaces using T4P‐mediated twitching motility (Wei et al. 2021; Kumar Verma et al. 2018, 2024). However, our data showed that the Xoc *emvR* deletion mutant had enhanced twitching motility but a reduced virulence on rice leaves with spraying inoculation. Given that the *emvR* deletion mutant displayed a weakened spreading motility observed on semisolid agar surfaces, it is possible that spreading motility is important for Xoc infection from rice stomata. Another possibility, but not mutually exclusive, is that the reduction in EPS production attenuates the growth and invasion of epiphytic Xoc. Although the *emvR* deletion mutant produces slightly less EPS compared with the wild type when cells are cultured in medium, it remains unknown whether EmvR significantly affects EPS production in bacteria grown on plant surfaces.

Like other *Xanthomonas* phytopathogens such as Xcc and Xcci (Zhang et al. 2008; Yan and Wang 2011), Xoc harbours three pairs of ColR/ColS homologues, ColS_XOCgx_1237_/ColR_XOCgx_1236_, ColS_XOCgx_3645_/ColR_XOCgx_3646_ and ColS_XOCgx_4036_/ColR_XOCgx_4037_ (Zhang et al. 2018). In Xcc and Xcci, only one (ColR_XC1049_/ColS_XC1050_ in Xcc and their counterparts in Xcci) out of three pairs of the ColR/ColS system has been characterised up to now, which is involved in virulence, hypersensitive reaction induction, bacterial growth and stress tolerance (Zhang et al. 2008; Yan and Wang 2011). The function of the other two ColR/ColS pairs is unknown yet. Xcc ColS_XC1050_/ColRXC1049 has the highest identity score with Xoc ColS_XOCgx_1237_/ColR_XOCgx_1236_ (96.1% and 100% for ColS_XOCgx_1237_ and ColR_XOCgx_1236_, respectively, while only 27.2% and 51.5% for ColS_XOCgx_4036_/ColR_XOCgx_4037_ and 23.4% and 50.8% for ColS_XOCgx_3645_/ColR_XOCgx_3646_). Our data showed that deletion of ColR_XOCgx_4037_ or ColS_XOCgx_4036_ weakened cell spreading but enhanced cell twitching (Figure 4). Interestingly, deletion of ColR_XOCgx_4037_ reduced EPS production, but deletion of ColS_XOCgx_4036_ did not (Figure 4). These observations imply that the TCS ColS_XOCgx_4036_/ColR_XOCgx_4037_ regulates twitching and spreading motility but not EPS production. The mechanism by which ColR_XOCgx_4037_ regulates EPS production needs to be further investigated.

Commonly, a typical TCS consists of an HK and an RR. The genes encoding both HK and RR are co‐located in the genome and co‐expressed; the two components usually interact in an exclusive one‐to‐one fashion (Stock et al. 2000; Gao et al. 2019). The genes encoding ColS_XOCgx_4036_ and ColR_XOCgx_4037_ are co‐located within the genome of Xoc, implying that ColS_XOCgx_4036_ and ColR_XOCgx_4037_ most probably constitute a TCS. In terms of amino acid sequence, ColR_XOCgx_4037_ is a typical RR, composed of 237 amino acids with a REC domain (residues 9–121) and a DNA‐binding domain (residues 155–229), suggesting that ColR_XOCgx_4037_ modulates spreading and twitching motility via binding to its target gene(s) that are involved directly or indirectly in motility.

Our co‐IP, B2H and protein pull‐down assays also demonstrated that EmvR interacts with the sensor HK ColS_XOCgx_4036_, suggesting that ColS_XOCgx_4036_ can also form another TCS with EmvR in addition to forming the TCS with ColR_XOCgx_4037_. We attempted to examine the phosphotransfer between ColS_XOCgx_4036_ and EmvR using the Phos‐tag method. However, no phosphotransfer was observed (data not shown). This could be due to the phosphorylation of EmvR from ColS_XOCgx_4036_ being too low to be detected by Phos‐tag, or the occurrence of phosphotransfer requiring certain unknown conditions. Previous studies have shown that there are many TCS architectures with two or more interacting HKs or RRs (for review, see Laub and Goulian 2007; Francis and Porter 2019). The signalling (or phosphorylation) pathway in these TCSs is branched, in a ‘one‐to‐many’ or a ‘many‐to‐one’ fashion. A number of TCSs with a ‘one‐to‐many’ fashion have been reported. One of the most well‐studied examples is the HK CheA that can phosphorylate the SD‐RR CheY and the RR CheB, which are members of the chemotaxis system of *E. coli* (Szurmant and Ordal 2004). Another well‐characterised ‘one‐to‐many’ HK is the Xcc HpaS, which controls multiple phenotypes via different RRs such as the RR HrpG and the SD‐RR VemR (Li et al. 2014, 2020; Li, Peng, et al. 2023). We supposed that under certain conditions, the sensor HK ColS_XOCgx_4036_ probably co‐opts the orphan SD‐RR EmvR to form a branched TCS that regulates PliB activity to modulate motility in Xoc.

**FIGURE 7:**
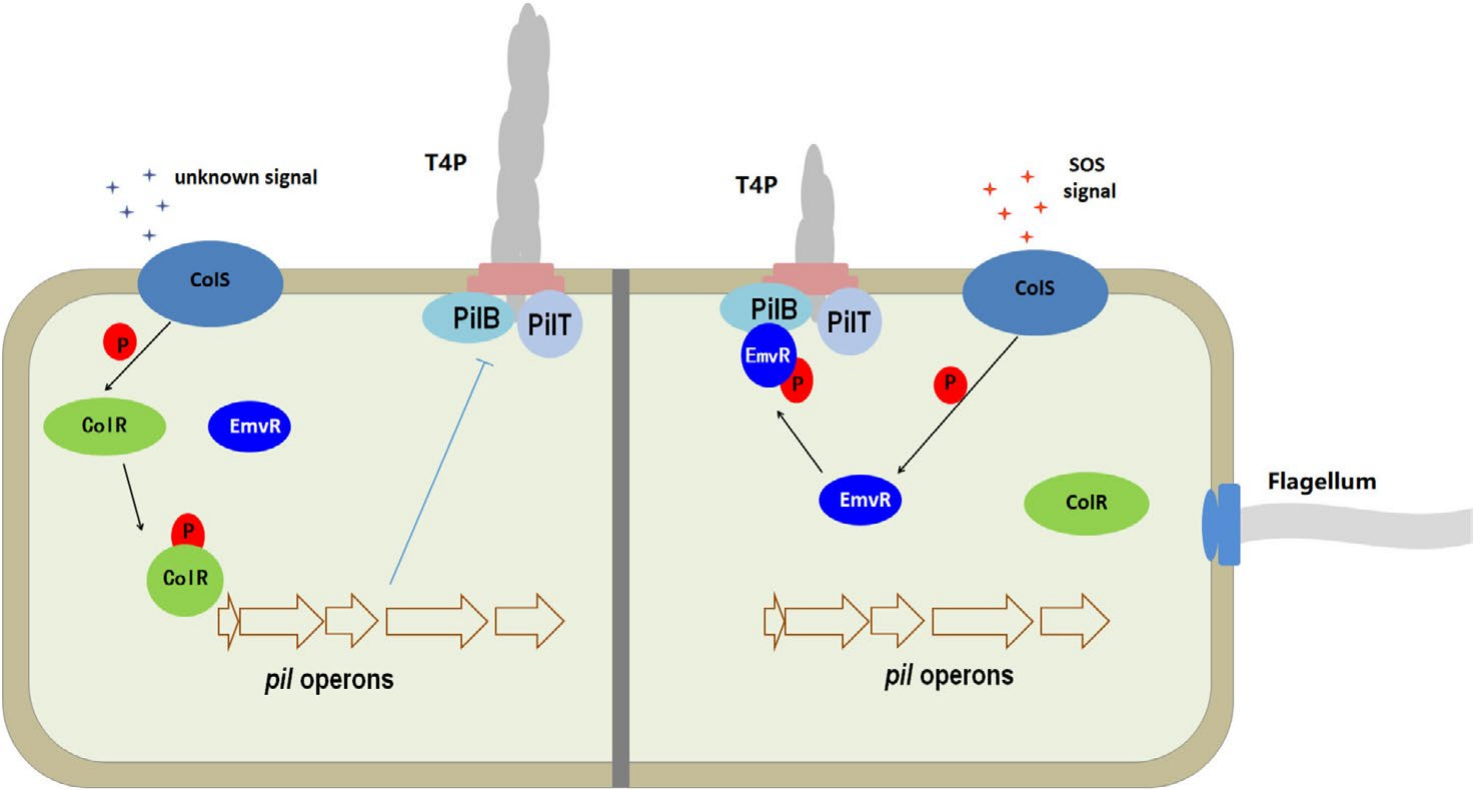
Working model of the role of the EmvR in type IV pilus (T4P)‐mediated motility. EmvR is co‐opted by sensor HK ColS_XOCgx_4036_ to form a branched two‐component system that modulates motility. When sensing an unknown environmental cue, ColS_XOCgx_4036_ autophosphorylates and transfers the phosphoryl group to the conserved aspartate residue in the REC domain of ColR_XOCgx_4037_, leading to a conformational change and the activation of its C‐terminal DNA‐binding domain, in turn causing a change in the expression levels of a set of genes, including motility‐related and extracellular polysaccharide (EPS)‐related genes, resulting in the repression of assembly/extension of T4P. When an emergency stimulus (extra‐ or intracellular signal) is present, ColS_XOCgx_4036_ autophosphorylates and then rapidly transfers the phosphoryl group to Asp‐59 of EmvR. This promotes the binding of EmvR to PilB, which reduces the ATPase activity of PilB. Then the assembly/extension of T4P is attenuated, leading to a reduction in twitching motility.

Growing evidence has revealed that some HKs can sense and respond to multiple signals in their different domains (Ishii and Eguchi 2021). Given that ColS_XOCgx_4036_ and ColR_XOCgx_4037_ control divergent phenotypes, maybe ColS_XOCgx_4036_ can sense and transfer different stimuli (including intracellular cues) in Xoc. In contrast to the PdeK/PdeR‐FimX/PilZ/PilB signalling pathway that activates the assembly/extension of T4P in Xoc and Xcci (Llontop et al. 2021; Wei et al. 2021), our data suggested that there is a regulatory pathway consisting of ColS_XOCgx_4036_, EmvR that represses the T4P assembly in Xoc. Here, we propose a working model for the role of ColS_XOCgx_4036_, ColR_XOCgx_4037_ and EmvR in T4P‐mediated motility (Figure 7). When sensing an unknown environmental cue, ColS_XOCgx_4036_ autophosphorylates and transfers the phosphoryl group to its cognate ColR_XOCgx_4037_, leading to a change in the expression levels of a set of genes directly or indirectly involved in T4P‐mediated motility. When an emergency stimulus is present, ColS_XOCgx_4036_ rapidly transfers the phosphoryl group to EmvR, which binds to PilB to repress its activity, thereby weakening T4P biogenesis.

## 4 Experimental Procedures

### 4.1 Bacterial Strains, Plasmids and Growth Conditions

Strains and plasmids used in this study are listed in Table S1. Xoc strains were grown at 28°C in NB medium (per litre:1 g yeast extract, 3 g beef extract, 5 g polypeptone, 10 g sucrose), or NA medium (NB with 15 g agar per litre). *E. coli* strains were grown in Luria–Bertani (LB) medium (per litre: 5 g yeast extract, 10 g NaCl, 10 g tryptone) or on LB agar plates (LB with 15 g agar per litre) at 37°C. Antibiotics were used at the following concentrations: kanamycin (Kan) at 25 μg/mL, rifampicin (Rif) at 50 μg/mL, ampicillin (Amp) at 100 μg/mL, spectinomycin (Spc) at 50 μg/mL, and tetracycline (Tet) at 5 μg/mL for *Xanthomonas* strains or 15 μg/mL for *E. coli* strains.

### 4.2 DNA and RNA Manipulations

The nucleic acid manipulations followed the procedures previously described (Sambrook et al. 1989). Conjugations between Xoc and *E. coli* strains were performed as previously described (Turner et al. 1985). The restriction endonucleases, T4 DNA ligase and Pfu polymerase (or GoTaq Green Master Mix) were provided by Promega. Total RNA was extracted from cultures of Xoc strains using a total RNA extraction kit (Invitrogen) and cDNA was generated using a cDNA synthesis kit (Invitrogen). To assay the transcription level of certain genes, RT‐qPCR was carried out as previously described (Li, Ren, et al. 2023) using the diluted cDNA as a template with selected primers for target genes. The relative mRNA level was calculated with respect to the level of the corresponding transcript in the wild‐type strain GX01 (equal to 1). The expression level of the 16S rRNA gene was used as an internal standard.

### 4.3 Construction of Mutant Strains and Reporter Strain With FLAG‐Tagged Protein

An in‐frame deletion mutant of *emvR*, *colS*_*XOCgx_4036*_, *colR*_*XOCgx_4037*_, or *pilB* in Xoc was constructed by using a method previously described (Li, Ren, et al. 2023). Briefly, upstream and downstream fragments of the *emvR*, *colS*_*XOCgx_4036*_, *colR*_*XOCgx_4037*_, or *pilB‐*coding region were PCR‐amplified using the corresponding primers listed in Table S3. The two fragments were cloned together into the vector pK18*mobsacB* (Table S1), and the obtained plasmid (pKΔ*emvR*, pKΔ*colS*_*XOCgx_4036*_, pKΔ*colR*_*XOCgx_4037*_ or pKΔ*pilB*) was introduced into the Xoc strain GX01 by triparental conjugation. The obtained mutants were named as Δ*emvR*, Δ*colS*_*XOCgx_4036*_, Δ*colR*_*XOCgx_4037*_ and Δ*pilB*, respectively (Table S1). For complementation of these mutants, the full length of *emvR* (432 bp), *colS*_*XOCgx_4036*_ (1609 bp), *colR*_*XOCgx_4037*_ (1183 bp) and *pilB* (1734 bp) were amplified by PCR from Xoc GX01 using the corresponding primers (Table S3) and cloned into the low‐copy number plasmid pXUK. The resulting plasmids named pXC*emvR*, pXC*colS*_*XOCgx_4036*_, pXC*colR*_*XOCgx_4037*_ and pXC*pilB* (Table S1) were introduced into Δ*emvR*, Δ*colS*_*XOCgx_4036*_, Δ*colR*_*XOCgx_4037*_ and Δ*pilB*, respectively, generating complemented strains CΔ*emvR*, CΔ*colS*_*XOCgx_4036*_, CΔ*colR*_*XOCgx_4037*_ and CΔ*pilB* (Table S1).

For cross‐complementation of the Δ*emvR* mutant, the recombinant plasmids pXC*colS*_*XOCgx_4036*_ and pXC*colR*_*XOCgx_4037*_ (Table S1) were transferred into the Δ*emvR* mutant by triparental conjugation resulting in strains Δ*emvR*/pXC*colS*_*XOCgx_4036*_ and Δ*emvR*/pXC*colR*_*XOCgx_4037*_. For cross‐complementation of the mutants Δ*colS*_*XOCgx_4036*_ and Δ*colR*_*XOCgx_4037*_, pXC*emvR* was introduced, and the obtained strains were named Δ*colS*_*XOCgx_4036*_/pXC*emvR* and Δ*colR*_*XOCgx_4037*_/pXC*emvR*, respectively.

For construction of a Xoc reporter strain chromosomally expressing EmvR fused with a 3 × FLAG‐tag (EmvR:3 × FLAG), a method as previously described (Li, Peng, et al. 2023) with minor modifications was used. Briefly, a 538‐bp DNA fragment (composed of 96‐bp DNA upstream of the *emvR* start codon, 402‐bp EmvR‐coding sequence and 40‐bp FLAG‐coding sequence) and a 549‐bp DNA fragment (composed of 46‐bp FLAG‐coding sequence, the 3‐bp stop codon of *emvR*, and 500‐bp downstream of the *emvR* stop codon) were generated by PCR amplification from the GX01 genomic DNA using the primer sets L*emvR*‐FlagF/R and R*emvR*‐FlagF/R (Table S3), respectively. The two fragments were joined using overlap extension PCR, and the resulting recombinant fragment was cloned into pK18mob*sacB* (Table S1). The resulting recombined plasmid named pK*emvR*::flag (Table S1) was introduced into Xoc GX01 via conjugation, and the variant strain chromosomally encoding EmvR:3 × FLAG protein was screened, confirmed, and named GX01(EmvR:3 × FLAG) (Table S1).

### 4.4 Site‐Directed Mutagenesis

Site‐directed mutagenesis of *emvR* was carried out using a method as described previously (Li et al. 2020). Briefly, *emvR* with codon substitution was created using a QuikChange II Site‐directed Mutagenesis kit (Stratagene) with recombinant plasmid pK*emvR* (containing the coding sequence of *emvR* gene) as template and the corresponding mutagenic oligonucleotides as primers (Table S3). Then the mutated gene was subcloned into the plasmid pK18mob*sacB* (Table S1). The obtained recombinant plasmids pK18*emvR*_*D15A*_, pK18*emvR*_*D59A*_ and pK18*emvR*_*T87A*_, in which the amino acids Asp‐15, Asp‐59 and Thr‐87 in EmvR were substituted by alanine, were separately transferred to Xoc GX01, and the transconjugants were screened on selective agar plates containing 5% sucrose. The obtained EmvR point mutant derivatives were named Δ*emvR*_*D15A*_, Δ*emvR*_*D59A*_ and Δ*emvR*_*T87A*_, respectively (Table S1).

### 4.5 Overexpression and Purification of Proteins

To obtain 6 × His‐tagged form of EmvR (6 × His:EmvR) and its variants EmvR_D15A_, EmvR_D59A_ and EmvR_T87A_, the 432‐bp innate and mutated *emvR‐*coding sequences were PCR‐amplified using primers *emvR*‐OF/R from the Xoc strains GX01, Δ*emvR*_*D15A*_, Δ*emvR*_*D59A*_ and Δ*emvR*_*T87A*_, respectively. The obtained DNA fragments were cloned into the expression vector pET‐30a. The resulting recombinant plasmids named pET‐30a‐EmvR, pET‐30a‐EmvR_D15A_, pET‐30a‐EmvR_D59A_ or pET‐30a‐EmvR_T87A_ (Table S1) were transformed into *E. coli* BL21. To overproduce 6 × His‐tagged form of PliB, PilT, PilO, PilU, as well as truncated ColS_XOCgx_4036_, the 1734‐bp *pilB*, 1035‐bp *pilT*, 666‐bp *pilO*, 1131‐bp *pilU* and 822‐bp *colS*_*XOCgx_4036*_ coding sequences were PCR‐amplified from Xoc GX01 using the corresponding primers listed in Table S3. The obtained DNA fragments were cloned into the expression vector pET‐30a to generate the recombinant plasmids pET‐30a‐PilB, pET‐30a‐PilT, pET‐30a‐PilO, pET‐30a‐PilU and pET‐30a‐ColS (Table S1). The recombinant plasmids were transformed into *E. coli* BL21 (DE3). The obtained strains were cultured and induced by isopropyl β‐D‐thiogalactopyranoside (IPTG), and then the cells were collected, and the fusion proteins were purified using Ni‐NTA resin (Qiagen).

### 4.6 Co‐Immunoprecipitation and LC–MS/MS Analysis

To identify EmvR‐interacting partners, co‐IP and LC–MS/MS analysis was performed as previously described (Li, Peng, et al. 2023), with minor modifications. Briefly, Xoc strains GX01 (EmvR:3 × FLAG) and GX01 (negative control) were cultured in NB medium overnight, and total proteins were prepared from the bacterial cells. The agarose‐conjugated anti‐FLAG was added and co‐incubated with the total protein sample. The eluted proteins from agarose beads were resolved by SDS‐PAGE and analysed by LC–MS/MS on a nano‐LC system combined with an LTQ‐Orbitrap Elite mass spectrometer. The MS data were analysed with SEQUEST against the Xoc Uniprot protein database (https://www.uniprot.org/taxonomy/383407). Peptides that were filtered with a confidence level of 99% were accepted. The FDR for the peptide was set to 1%.

### 4.7 Bacterial Two‐Hybrid Assay

The BacterioMatch II two‐hybrid system (Stratagene) was used to detect EmvR–protein interactions. Analysis was carried out according to the manufacturer's instructions and previously described (Li et al. 2020). Briefly, a DNA fragment of the *emvR* gene, which was obtained by PCR amplification from Xoc GX01 using the primer set *emvR*‐BTF/R (Table S3), was cloned into the bait vector pBT, generating the plasmid pBT*emvR*. Simultaneously, DNA fragments of *pilB*, *pilT*, *colS*_*XOCgx_4036*_ and other selected genes were PCR amplified using the corresponding primers listed in Table S3 and cloned into the target vector pTRG, resulting in plasmids pTRG*pilB*, pTRG*pilT*, pTRG*colS*_*XOCgx_4036*_ and so on (Table S1). The *E. coli* XL1‐Blue MRFʹ reporter strains harbouring different plasmid pairs were grown on the nonselective plates and double‐selective indicator plates containing 5 mM 3‐amino‐1,2,4‐triazole (3‐AT) and 12.5 μg/mL streptomycin (Sm).

### 4.8 Protein Pull‐Down Assay

Protein pull‐down assays were performed as previously described (Li et al. 2014), with the ProFound pull‐down biotinylated protein–protein interaction kit (Pierce). Briefly, 6 × His:EmvR was biotinylated with sulfo‐NHS‐LC‐biotin. Then 50 μL of the purified biotinylated 6 × His:EmvR (0.5 mg/mL) was incubated with streptavidin Sepharose beads. After washing, the beads were incubated with samples containing suspected prey proteins 6 × His:PilB, 6 × His:PilO, 6 × His:PilU, 6 × His:PilT, or 6 × His:ColS_XOCgx_4036_. Then the beads were washed and the prey protein was eluted using elution buffer (pH 2.8). Twenty microlitres of the eluted sample was electrophoresed on a 12% SDS‐PAGE gel and visualised by Coomassie blue staining.

### 4.9 ATPase Assay

The ATPase activity of PilB was analysed using the malachite green phosphate detection kit (Beyotime). Briefly, 2 μg of PilB protein was used for each reaction. The assay was carried out in reaction buffer (25 mM Tris–HCl pH 7.0, 50 mM NaCl, 2 mM MgCl_2_, 2 mM β‐mercaptoethanol) and 1 mM ATP (or a range of ATP concentrations), and incubated for 30 min at 30°C. An aliquot (200 μL) of each reaction was transferred to a 96‐well plate and 70 μL of freshly prepared phosphate assay reagent was added. After 30 min of incubation at room temperature, the absorbance was measured at 630 nm using a 96‐well microplate reader. Reactions lacking PilB or ATP were used as negative controls. The phosphate released during the reaction was measured using a standard curve prepared with 0.1 to 10 nM phosphate (KH_2_PO_4_).

### 4.10 Western Blotting

Western blotting followed the procedure described by Sambrook et al. (1989). Bacterial proteins separated by SDS‐PAGE were electrotransferred onto a polyvinylidene difluoride (PVDF) membrane (Millipore). After blocking with 1% milk, the proteins in the membrane were incubated with the 1:2500 diluted anti‐FLAG‐tag mouse monoclonal antibody (Solarbio) as the primary antibody, followed by washing with Tris‐buffered saline with Tween buffer (20 mM Tris, 0.3 M NaCl, 0.08% Tween 20). The diluted 1:2500 horseradish peroxidase (HRP)‐conjugated goat anti‐mouse immunoglobulin G (IgG) was used as the secondary antibody. The luminescence signal was detected according to the manufacturer's instructions. For a loading control, proteins were probed with the anti‐RNAP β‐antibody (Abcam) at a 1:2000 dilution as the primary antibody, and the HRP‐conjugated goat anti‐rabbit IgG HandL (Thermo Scientific) at a 1:5000 dilution as the secondary antibody.

### 4.11 In Vitro Phosphorylation Assay

The phosphorylation of EmvR and its variants in vitro was analysed using a biotinylated phosphate binding tag Phos‐tag BTL‐104 (Wako Pure Chemical Industries Ltd). The experiment was carried out according to the manufacturer's instructions and previously described (Kinoshita et al. 2012) with slight modifications. Briefly, 3 μg of the purified proteins (6 × His:EmvR, and its variants EmvR_D15A_, EmvR_D59A_ and EmvR_T87A_) were incubated with 50 mM lithium potassium acetyl phosphate (AcP) (Sigma) at 37°C for 30 min in a buffer (40 mM Tris–HCl, pH 8.0, 10 mM MgCl_2_, 40 mM KCl, 1 mM dithiothreitol), then samples were run on SDS‐PAGE gels, after which the proteins were transferred to PVDF membranes. The membrane was blocked with 10% (wt/vol) bovine serum albumen (BSA), followed by incubation with a complex of Phos‐tag BTL‐104 with streptavidin‐conjugated horseradish peroxidase (HRP‐SA) to detect protein phosphorylation. For loading control, equal amounts of proteins were run on an SDS‐PAGE gel and then transferred onto a PVDF membrane. After being blocked with BSA, the membrane was treated with anti‐His antibody as the primary antibody and HRP‐conjugated goat anti‐mouse immunoglobulin G (IgG) as the secondary antibody.

### 4.12 EPS and Enzyme Assays

EPS assays were performed as previously described (Tang et al. 1991) with minor modifications. Briefly, to estimate EPS production, two microlitres of suspension (10^9^ cfu/mL) of each Xoc strain was spotted onto NA with 2% sucrose and grown for 5 days. For quantification of EPS production, Xoc strains were cultured in 100 mL NB broth containing 4% sucrose at 28°C with shaking at 200 rpm for 3 days. EPS was precipitated from the culture supernatant with ethanol, then dried and weighed.

### 4.13 Bacterial Motility Assays

The swimming and spreading motilities of Xoc strains were detected as previously described (Li et al. 2020). Two micolitres of bacterial suspension (10^9^ cfu/mL) was stabbed into 0.28% (wt/vol) agar plates composed of 0.03% Bacto peptone and 0.03% yeast extract (for swimming) or spotted on NA plates containing 2% glucose and 0.6% (wt/vol) agar (for spreading). The diameters of the area occupied by the bacterial cells were measured after 4–5 days of incubation at 28°C. The subsurface twitching motility (twitching motility between the agar and Petri dish interface) of Xoc strains was tested using the stab‐inoculation method as previously described (Dunger et al. 2014) with some modifications. Briefly, two micolitres of bacterial suspension (10^9^ cfu/mL) was stabbed through a new NA plate (1% wt/vol agar) to the plastic surface, and growth occurred for 5–7 days. Upon removal of the agar, the zone of twitching motility was visualised by staining with 0.1% (wt/vol) crystal violet, and the unbound dye was removed by rinsing with ultrapure water. The twitching areas were photographed and calculated using ImageJ. The experiments were repeated independently three times.

### 4.14 Virulence Tests

Virulence tests were performed on greenhouse‐grown 6‐week‐old rice plants of the lines IRBB5 and IRBB21 by syringe infiltrating and spraying methods as previously described (Li, Ren, et al. 2023), with minor modifications. Briefly, bacterial cells were collected from cultures of the Xoc wild‐type strain and its derivatives and resuspended in sterile water to a concentration of OD_600_ of 0.3 (~10^8^ cfu/mL). The resuspended bacterial cells were inoculated into rice leaves by infiltrating with a needleless syringe, and the resulting lesions and symptoms were evaluated 10 days post‐inoculation. For the spraying method, ~ 50 leaves were sprayed with a volume of 20 mL of the bacterial suspensions. Three replicates of each independent experiment were carried out. Ten days after inoculation, the relative virulence was determined by calculating the percentage of the total inoculated leaves (~50 leaves) that showed typical BLS symptoms. The experiment was repeated three times.

## Conflicts of Interest

The authors declare no conflicts of interest.

## Supporting information


**Figure S1.** EmvR has not influence on activity of extracellular enzymes. Plate assays (Tang et al. 1991) were employed to qualitatively test extracellular enzyme activities. A bacterial culture (2 μL) of each *Xanthomonas oryzae* pv. *oryzicola* (Xoc) strain was spotted onto ‘protease’ plates, ‘endoglucanase’ plates or ‘amylase’ plates and incubated at 28°C for 2 days. The *emvR* deletion mutant strain Δ*emvR* exhibited similar sizes of clearance zone around the inoculation spot on ‘protease’, ‘endoglucanase’ or ‘amylase’ plates, compared with the wild‐type strain.

**Figure S2.** Overexpression of EmvR has not influence on swimming motility and activity of extracellular enzymes. A bacterial culture (2 μL) of each *Xanthomonas oryzae* pv. *oryzicola* (Xoc) strain was stabbed into ‘swimming’ plates, or spotted onto ‘protease’ plates, ‘endoglucanase’ plates or ‘amylase’ plates and incubated at 28°C for 2–3 days. No differences were seen between the overexpression strain GX01/pX*emvR* and the wild‐type on all the tested plates.

**Figure S3.** Western blot of the eluted EmvR:3 × FLAG fusion protein. *Xanthomonas oryzae* pv. *oryzicola* (Xoc) wild‐type strain GX01 (negative control) and reporter strain GX01(EmvR:3 × FLAG) were cultured in NB medium, and total proteins were prepared. After co‐immunoprecipitation, protein samples were separated by SDS‐PAGE and transferred to a polyvinylidene difluoride (PVDF) membrane. The presence of the fusion proteins was detected by an anti‐FLAG‐tag mouse monoclonal antibody.

**Figure S4.** Bacterial two‐hybrid experiment testing the interactions of EmvR with the methyl‐accepting chemotaxis proteins (MCPs) XOCgx_2212, XOCgx_2487, XOCgx_2601, XOCgx_2603, XOCgx_2604 and XOCgx_2606. The reporter strains *Escherichia coli* XL1‐blue MRF′ with different plasmid pairs were inoculated on nonselective plates and double‐selection indicator plates (inoculated with a cell concentration of OD_600_ = 1.0) containing 3‐amino‐1, 2, 4‐triazole (3‐AT) and streptomycin (Sm). The reporter strains expressing EmvR and tested individual MCP were incapable of growth on the screening medium. Positive control strain grew well on the same screening medium. Three independent experiments showed similar results.

**Figure S5.** Overexpression of the *colS*_*XOCgx_4036*_ or *colR*_*xocgx_4037*_ gene in the *Xanthomonas oryzae* pv. *oryzicola* (Xoc) *emvR* deletion mutant cannot restore its spreading motility and extracellular polysaccharide (EPS) production. Two microlitres of culture suspensions (10^9^ cfu/mL) of Xoc wild‐type strain GX01, *emvR* deletion mutant Δ*emvR* and cross‐complemented strains Δ*emvR*/pXC*colS*_*XOCgx_4036*_ and Δ*emvR*/pXC*colR*_*XOCgx_4037*_ were inoculated onto ‘spreading’ plates (a) and NA plates containing 2% sucrose (b), and incubated for 5 days at 28°C. The strains Δ*emvR*/pXC*colS*_*XOCgx_4036*_ and Δ*emvR*/pXC*colR*_*XOCgx_4037*_ displayed similar colonies to the Δ*emvR* mutant.

**Figure S6.** Mutation in *pilB* reduces twitching motility but enhances spreading motility in *Xanthomonas oryzae* pv. *oryzicola* (Xoc). The Xoc wild‐type strain GX01, *pilB* deletion mutant Δ*pilB*, and complemented strain CΔ*pilB* were inoculated into/on ‘twitching’ and ‘spreading’ plates, respectively, and grown for 5 days. When necessary, agar was removed and the Petri plate surface was stained with crystal violet. The representative colony morphologies of Xoc strains were photographed.

**Figure S7.** Examination of the transcript level of *emvR* and its derivatives in *Xanthomonas oryzae* pv. *oryzicola* (Xoc) strains using reverse transcription‐quantitative PCR (RT‐qPCR) assay. The Xoc strain wild‐type strain GX01 and point‐mutated strains Δ*emvR*_*D15A*_, Δ*emvR*_*D59A*_ and Δ*emvR*_*T87A*_ were cultured in NB medium, and RNAs were extracted. The RT‐qPCR tests were performed in triplicate. Values given are the mean ± SD from triplicate measurements in a representative experiment.

**Table S1.** Bacterial strains and plasmids used in this work.

**Table S2.** The potential EmvR‐interacting proteins identified by co‐immunoprecipitation coupled with liquid chromatography–tandem‐mass spectrometry (LC–MS/MS) assays.

**Table S3.** Primers used in this work.

## Data Availability

The data that support the findings of this study are available from the corresponding author upon reasonable request.
