# Supplementary material for: Xanthomonas oryzae Orphan Response Regulator EmvR Is Involved in Virulence, Extracellular Polysaccharide Production and Cell Motility
Source: Mol Plant Pathol. 2025 Apr 6;26(4):e70083. doi: 10.1111/mpp.70083 (PMC11973254; doi:10.1111/mpp.70083)
Supplement: Supplementary file 1 — Figure S1. EmvR has not influence on activity of extracellular enzymes. Plate assays (Tang et al. 1991) were employed to qualitatively test extracellular enzyme activities. A bacterial culture (2 μL) of each Xanthomonas oryzae pv. oryzicola (Xoc) strain was spotted onto ‘protease’ plates, ‘endoglucanase’ plates or ‘amylase’ plates and incubated at 28°C for 2 days. The emvR deletion mutant strain ΔemvR exhibited similar sizes of clearance zone around the inoculation spot on ‘protease’, ‘endoglucanase’ or ‘amylase’ plates, compared with the wild‐type strain. [file MPP-26-e70083-s001.pptx]

## Slide 1
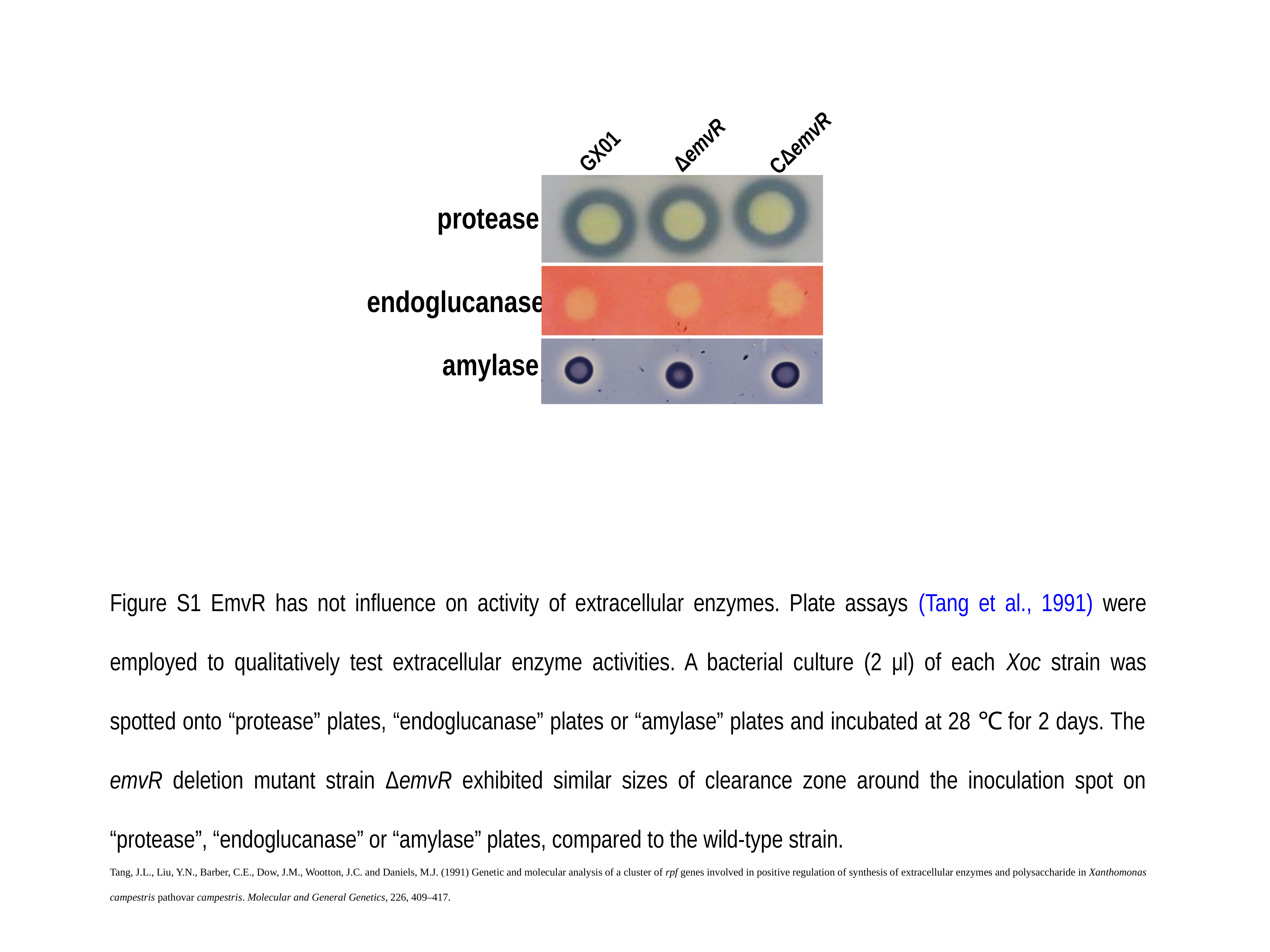

CΔemvR
ΔemvR
GX01
protease
endoglucanase
amylase
Figure S1 EmvR has not influence on activity of extracellular enzymes. Plate assays (Tang et al., 1991) were employed to qualitatively test extracellular enzyme activities. A bacterial culture (2 μl) of each Xoc strain was spotted onto “protease” plates, “endoglucanase” plates or “amylase” plates and incubated at 28 ℃ for 2 days. The emvR deletion mutant strain ΔemvR exhibited similar sizes of clearance zone around the inoculation spot on “protease”, “endoglucanase” or “amylase” plates, compared to the wild-type strain.
Tang, J.L., Liu, Y.N., Barber, C.E., Dow, J.M., Wootton, J.C. and Daniels, M.J. (1991) Genetic and molecular analysis of a cluster of rpf genes involved in positive regulation of synthesis of extracellular enzymes and polysaccharide in Xanthomonas campestris pathovar campestris. Molecular and General Genetics, 226, 409–417.
