# Supplementary material for: Xanthomonas oryzae Orphan Response Regulator EmvR Is Involved in Virulence, Extracellular Polysaccharide Production and Cell Motility
Source: Mol Plant Pathol. 2025 Apr 6;26(4):e70083. doi: 10.1111/mpp.70083 (PMC11973254; doi:10.1111/mpp.70083)
Supplement: Supplementary file 2 — Figure S2. Overexpression of EmvR has not influence on swimming motility and activity of extracellular enzymes. A bacterial culture (2 μL) of each Xanthomonas oryzae pv. oryzicola (Xoc) strain was stabbed into ‘swimming’ plates, or spotted onto ‘protease’ plates, ‘endoglucanase’ plates or ‘amylase’ plates and incubated at 28°C for 2–3 days. No differences were seen between the overexpression strain GX01/pXemvR and the wild‐type on all the tested plates. [file MPP-26-e70083-s005.pptx]

## Slide 1
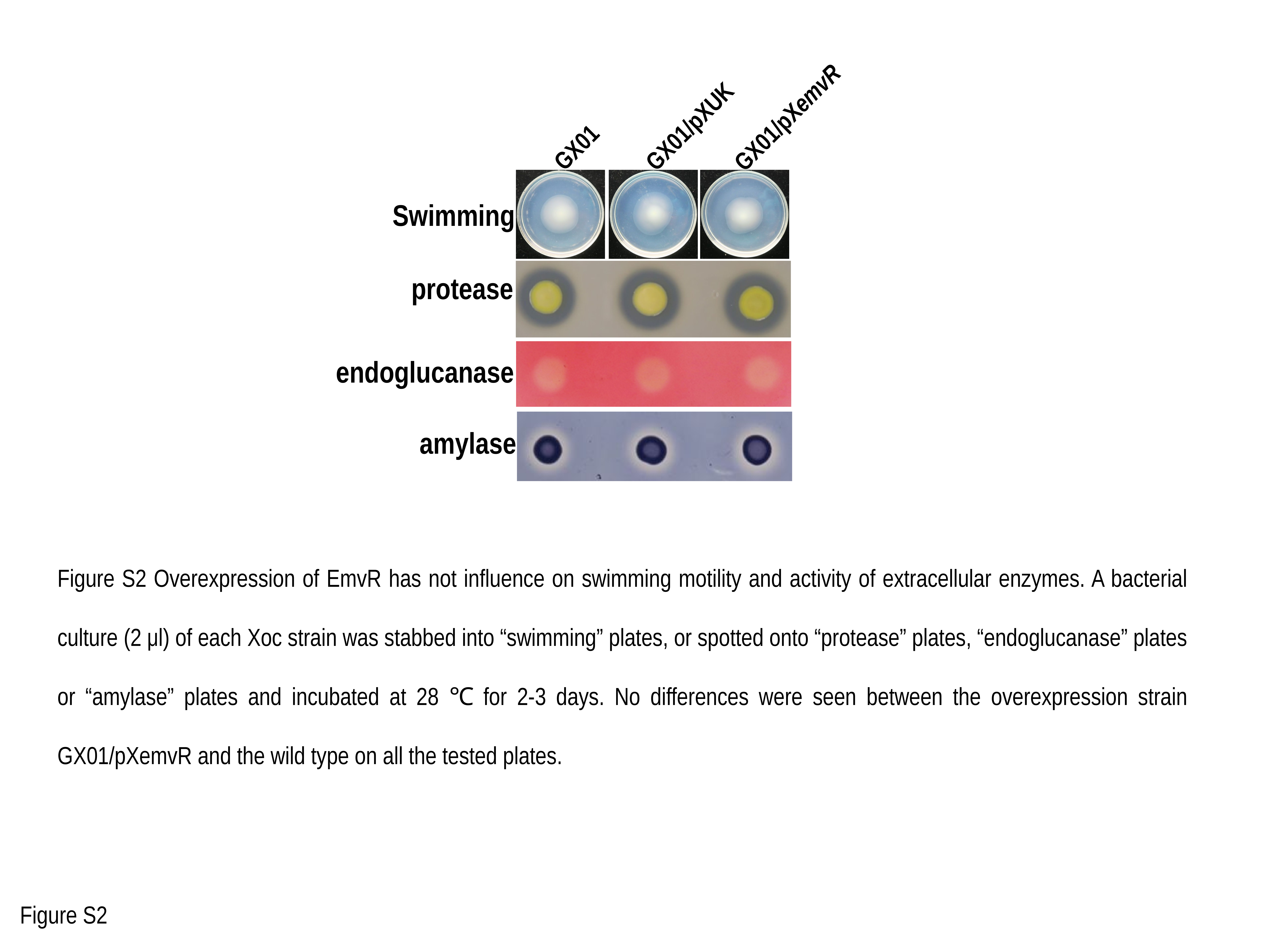

GX01/pXemvR
GX01/pXUK
GX01
Swimming
protease
endoglucanase
amylase
Figure S2 Overexpression of EmvR has not influence on swimming motility and activity of extracellular enzymes. A bacterial culture (2 μl) of each Xoc strain was stabbed into “swimming” plates, or spotted onto “protease” plates, “endoglucanase” plates or “amylase” plates and incubated at 28 ℃ for 2-3 days. No differences were seen between the overexpression strain GX01/pXemvR and the wild type on all the tested plates.
Figure S2
