# Supplementary material for: Xanthomonas oryzae Orphan Response Regulator EmvR Is Involved in Virulence, Extracellular Polysaccharide Production and Cell Motility
Source: Mol Plant Pathol. 2025 Apr 6;26(4):e70083. doi: 10.1111/mpp.70083 (PMC11973254; doi:10.1111/mpp.70083)
Supplement: Supplementary file 3 — Figure S3. Western blot of the eluted EmvR:3 × FLAG fusion protein. Xanthomonas oryzae pv. oryzicola (Xoc) wild‐type strain GX01 (negative control) and reporter strain GX01(EmvR:3 × FLAG) were cultured in NB medium, and total proteins were prepared. After co‐immunoprecipitation, protein samples were separated by SDS‐PAGE and transferred to a polyvinylidene difluoride (PVDF) membrane. The presence of the fusion proteins was detected by an anti‐FLAG‐tag mouse monoclonal antibody. [file MPP-26-e70083-s003.pptx]

## Slide 1
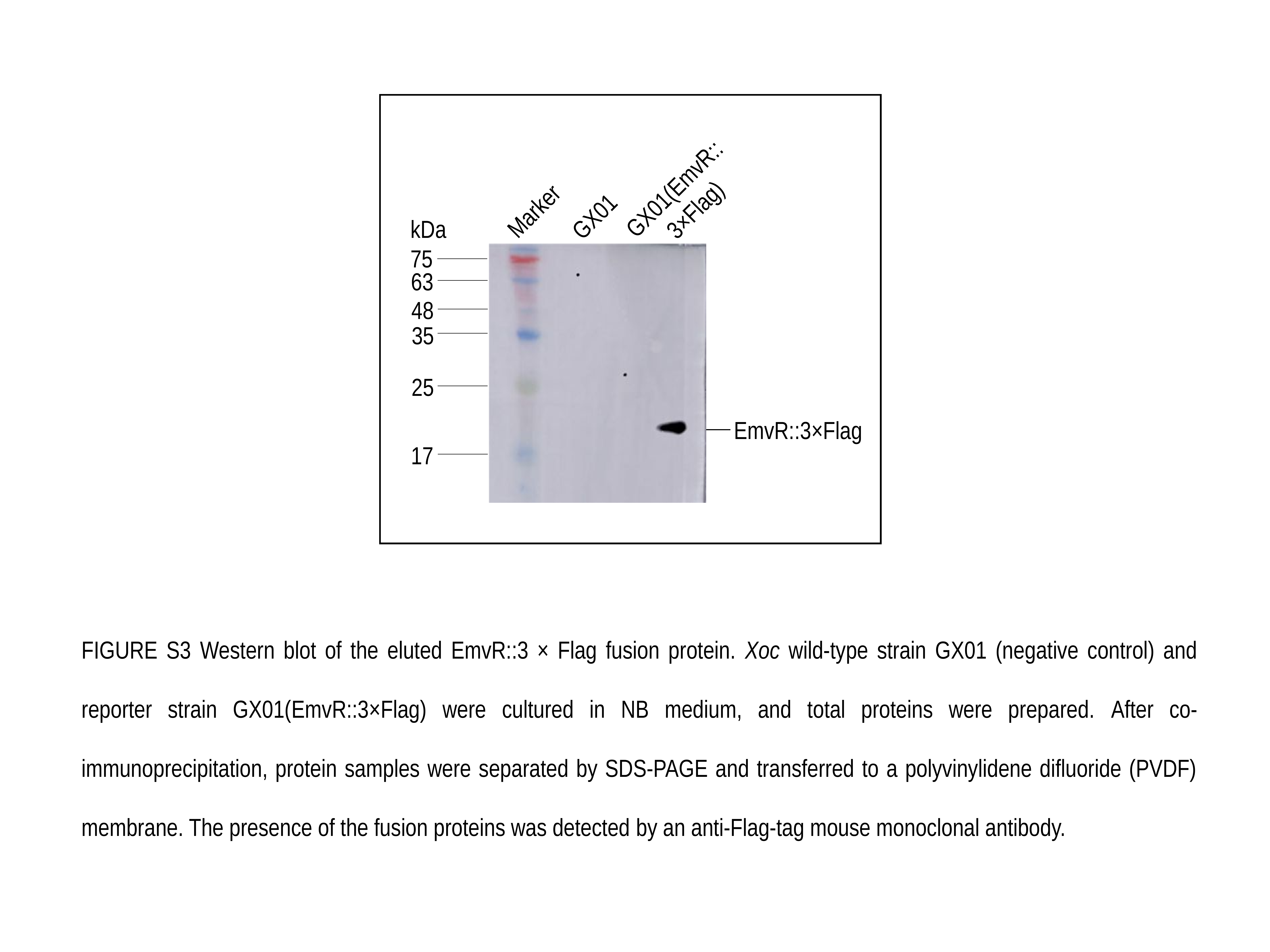

GX01(EmvR::3×Flag)
Marker
GX01
kDa
75
63
48
35
25
17
EmvR::3×Flag
FIGURE S3 Western blot of the eluted EmvR::3 × Flag fusion protein. Xoc wild-type strain GX01 (negative control) and reporter strain GX01(EmvR::3×Flag) were cultured in NB medium, and total proteins were prepared. After co-immunoprecipitation, protein samples were separated by SDS-PAGE and transferred to a polyvinylidene difluoride (PVDF) membrane. The presence of the fusion proteins was detected by an anti-Flag-tag mouse monoclonal antibody.
