# Supplementary material for: Xanthomonas oryzae Orphan Response Regulator EmvR Is Involved in Virulence, Extracellular Polysaccharide Production and Cell Motility
Source: Mol Plant Pathol. 2025 Apr 6;26(4):e70083. doi: 10.1111/mpp.70083 (PMC11973254; doi:10.1111/mpp.70083)
Supplement: Supplementary file 4 — Figure S4. Bacterial two‐hybrid experiment testing the interactions of EmvR with the methyl‐accepting chemotaxis proteins (MCPs) XOCgx_2212, XOCgx_2487, XOCgx_2601, XOCgx_2603, XOCgx_2604 and XOCgx_2606. The reporter strains Escherichia coli XL1‐blue MRF′ with different plasmid pairs were inoculated on nonselective plates and double‐selection indicator plates (inoculated with a cell concentration of OD600 = 1.0) containing 3‐amino‐1, 2, 4‐triazole (3‐AT) and streptomycin (Sm). The reporter strains expressing EmvR and tested individual MCP were incapable of growth on the screening medium. Positive control strain grew well on the same screening medium. Three independent experiments showed similar results. [file MPP-26-e70083-s007.pptx]

## Slide 1
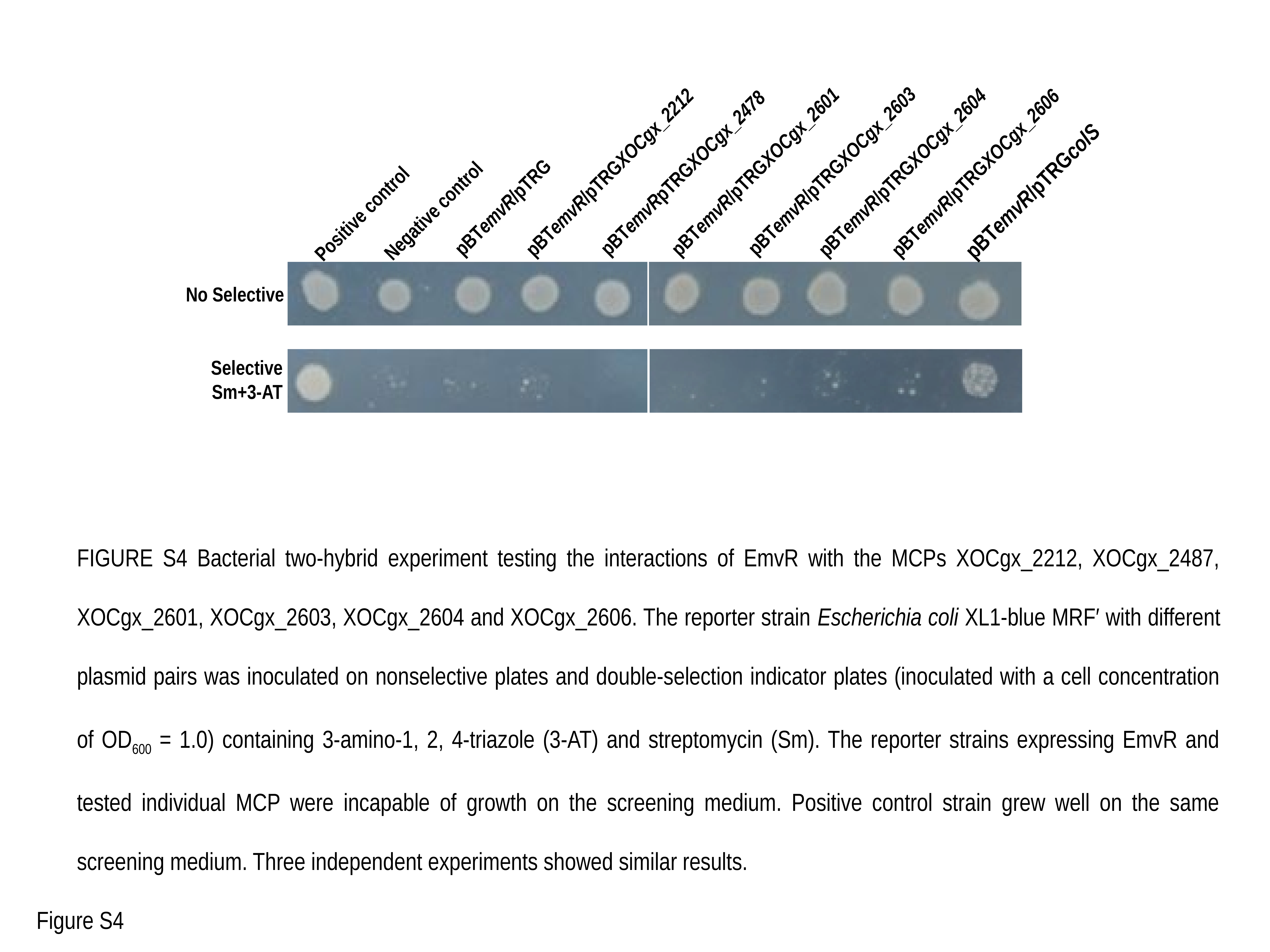

pBTemvR/pTRGXOCgx_2606
pBTemvR/pTRGXOCgx_2603
pBTemvRpTRGXOCgx_2478
pBTemvR/pTRGXOCgx_2604
pBTemvR/pTRGXOCgx_2601
pBTemvR/pTRGXOCgx_2212
pBTemvR/pTRGcolS
pBTemvR/pTRG
Negative control
Positive control
No Selective
 Selective
Sm+3-AT
FIGURE S4 Bacterial two-hybrid experiment testing the interactions of EmvR with the MCPs XOCgx_2212, XOCgx_2487, XOCgx_2601, XOCgx_2603, XOCgx_2604 and XOCgx_2606. The reporter strain Escherichia coli XL1-blue MRF′ with different plasmid pairs was inoculated on nonselective plates and double-selection indicator plates (inoculated with a cell concentration of OD600 = 1.0) containing 3-amino-1, 2, 4-triazole (3-AT) and streptomycin (Sm). The reporter strains expressing EmvR and tested individual MCP were incapable of growth on the screening medium. Positive control strain grew well on the same screening medium. Three independent experiments showed similar results.
Figure S4
