# Supplementary material for: Xanthomonas oryzae Orphan Response Regulator EmvR Is Involved in Virulence, Extracellular Polysaccharide Production and Cell Motility
Source: Mol Plant Pathol. 2025 Apr 6;26(4):e70083. doi: 10.1111/mpp.70083 (PMC11973254; doi:10.1111/mpp.70083)
Supplement: Supplementary file 5 — Figure S5. Overexpression of the colS XOCgx_4036 or colR xocgx_4037 gene in the Xanthomonas oryzae pv. oryzicola (Xoc) emvR deletion mutant cannot restore its spreading motility and extracellular polysaccharide (EPS) production. Two microlitres of culture suspensions (109 cfu/mL) of Xoc wild‐type strain GX01, emvR deletion mutant ΔemvR and cross‐complemented strains ΔemvR/pXCcolS XOCgx_4036 and ΔemvR/pXCcolR XOCgx_4037 were inoculated onto ‘spreading’ plates (a) and NA plates containing 2% sucrose (b), and incubated for 5 days at 28°C. The strains ΔemvR/pXCcolS XOCgx_4036 and ΔemvR/pXCcolR XOCgx_4037 displayed similar colonies to the ΔemvR mutant. [file MPP-26-e70083-s009.pptx]

## Slide 1
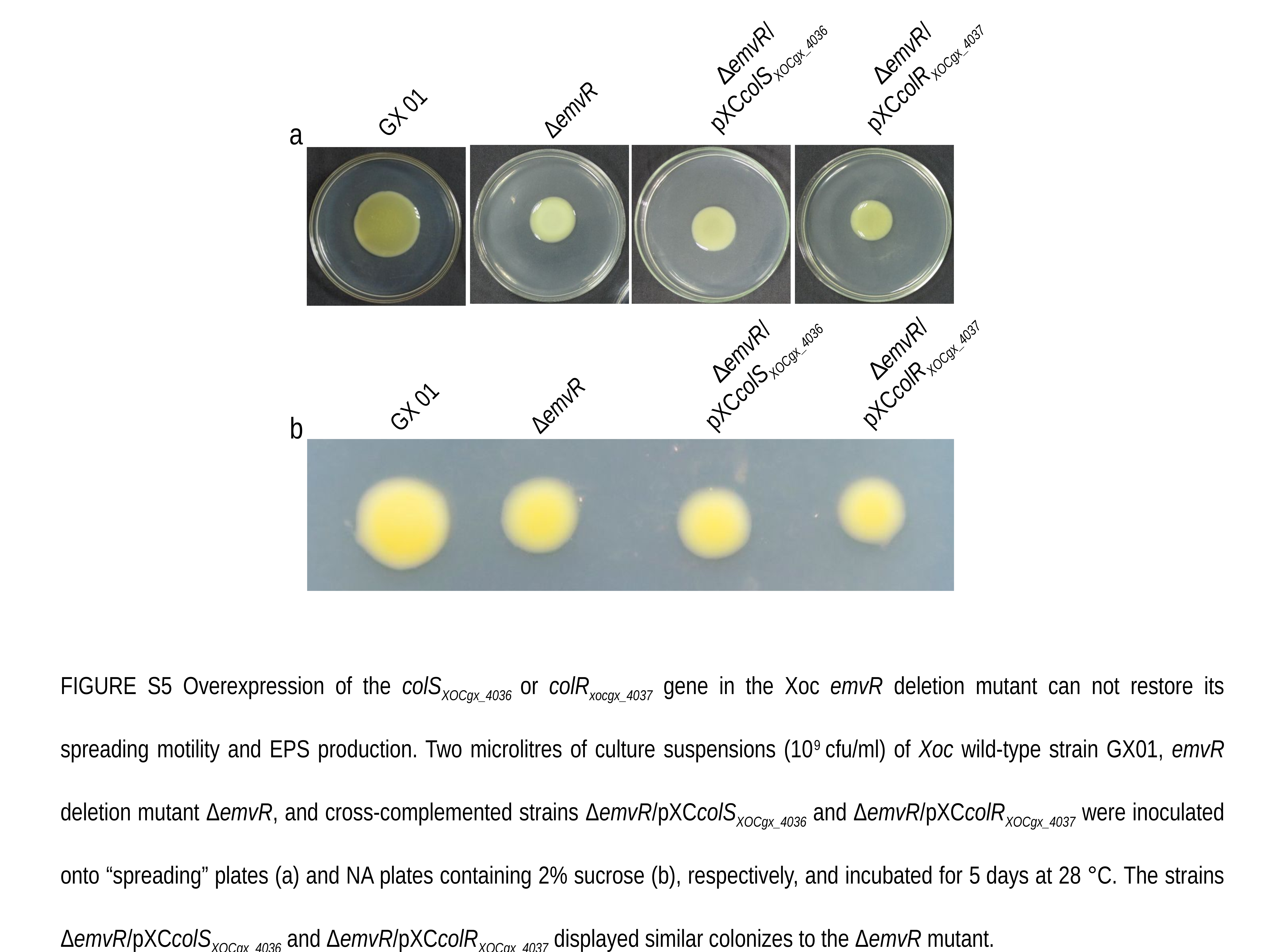

∆emvR/
pXCcolRXOCgx_4037
∆emvR/
pXCcolSXOCgx_4036
ΔemvR
GX 01
a
∆emvR/
pXCcolRXOCgx_4037
∆emvR/
pXCcolSXOCgx_4036
ΔemvR
GX 01
b
FIGURE S5 Overexpression of the colSXOCgx_4036 or colRxocgx_4037 gene in the Xoc emvR deletion mutant can not restore its spreading motility and EPS production. Two microlitres of culture suspensions (109 cfu/ml) of Xoc wild-type strain GX01, emvR deletion mutant ΔemvR, and cross-complemented strains ΔemvR/pXCcolSXOCgx_4036 and ΔemvR/pXCcolRXOCgx_4037 were inoculated onto “spreading” plates (a) and NA plates containing 2% sucrose (b), respectively, and incubated for 5 days at 28 °C. The strains ΔemvR/pXCcolSXOCgx_4036 and ΔemvR/pXCcolRXOCgx_4037 displayed similar colonizes to the ΔemvR mutant.
