# Supplementary material for: Xanthomonas oryzae Orphan Response Regulator EmvR Is Involved in Virulence, Extracellular Polysaccharide Production and Cell Motility
Source: Mol Plant Pathol. 2025 Apr 6;26(4):e70083. doi: 10.1111/mpp.70083 (PMC11973254; doi:10.1111/mpp.70083)
Supplement: Supplementary file 6 — Figure S6. Mutation in pilB reduces twitching motility but enhances spreading motility in Xanthomonas oryzae pv. oryzicola (Xoc). The Xoc wild‐type strain GX01, pilB deletion mutant ΔpilB, and complemented strain CΔpilB were inoculated into/on ‘twitching’ and ‘spreading’ plates, respectively, and grown for 5 days. When necessary, agar was removed and the Petri plate surface was stained with crystal violet. The representative colony morphologies of Xoc strains were photographed. [file MPP-26-e70083-s006.pptx]

## Slide 1
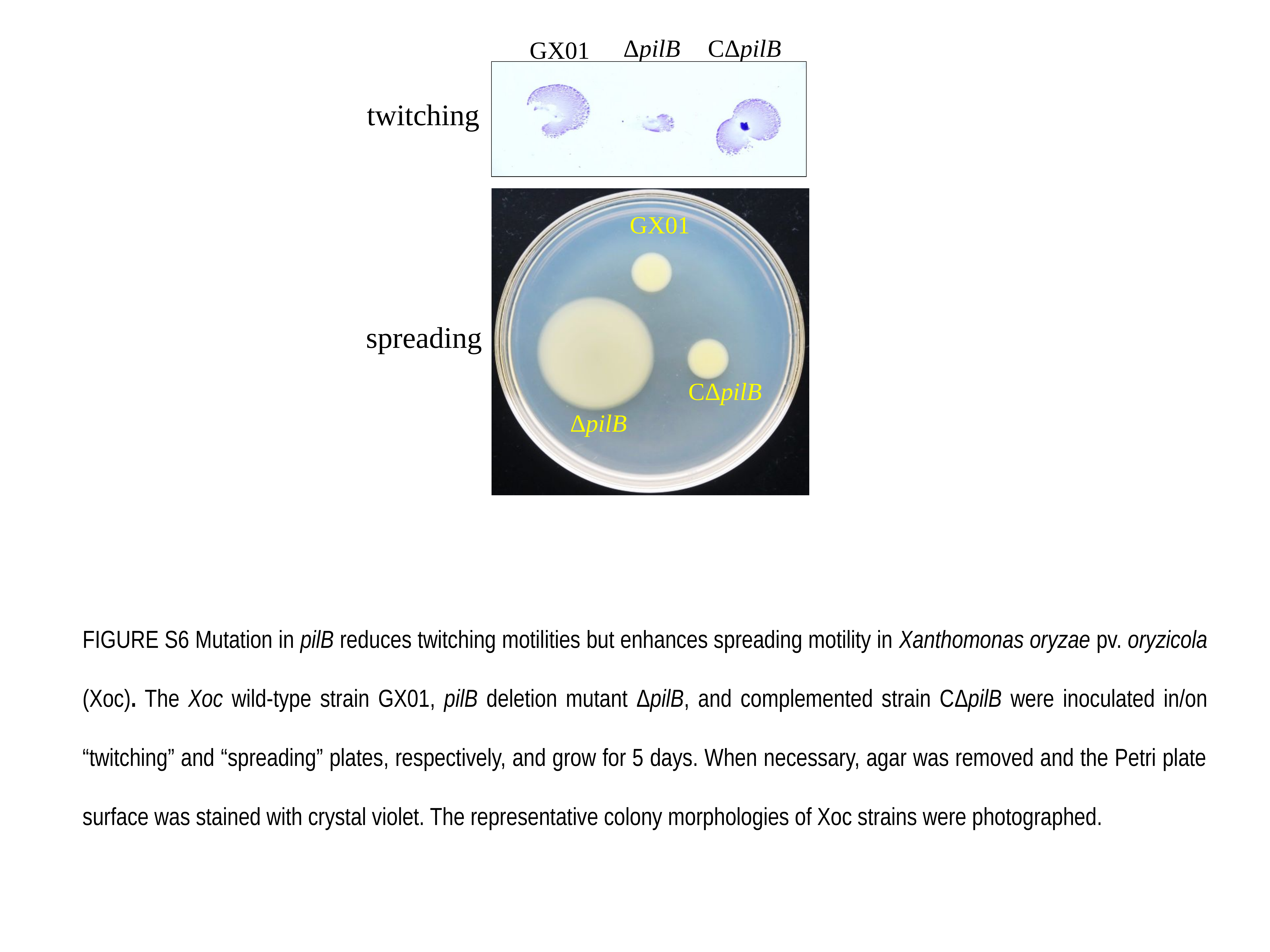

ΔpilB
CΔpilB
GX01
twitching
GX01
CΔpilB
ΔpilB
spreading
FIGURE S6 Mutation in pilB reduces twitching motilities but enhances spreading motility in Xanthomonas oryzae pv. oryzicola (Xoc). The Xoc wild-type strain GX01, pilB deletion mutant ΔpilB, and complemented strain CΔpilB were inoculated in/on “twitching” and “spreading” plates, respectively, and grow for 5 days. When necessary, agar was removed and the Petri plate surface was stained with crystal violet. The representative colony morphologies of Xoc strains were photographed.
