# Supplementary material for: Xanthomonas oryzae Orphan Response Regulator EmvR Is Involved in Virulence, Extracellular Polysaccharide Production and Cell Motility
Source: Mol Plant Pathol. 2025 Apr 6;26(4):e70083. doi: 10.1111/mpp.70083 (PMC11973254; doi:10.1111/mpp.70083)
Supplement: Supplementary file 7 — Figure S7. Examination of the transcript level of emvR and its derivatives in Xanthomonas oryzae pv. oryzicola (Xoc) strains using reverse transcription‐quantitative PCR (RT‐qPCR) assay. The Xoc strain wild‐type strain GX01 and point‐mutated strains ΔemvR D15A , ΔemvR D59A and ΔemvR T87A were cultured in NB medium, and RNAs were extracted. The RT‐qPCR tests were performed in triplicate. Values given are the mean ± SD from triplicate measurements in a representative experiment. [file MPP-26-e70083-s002.pptx]

## Slide 1
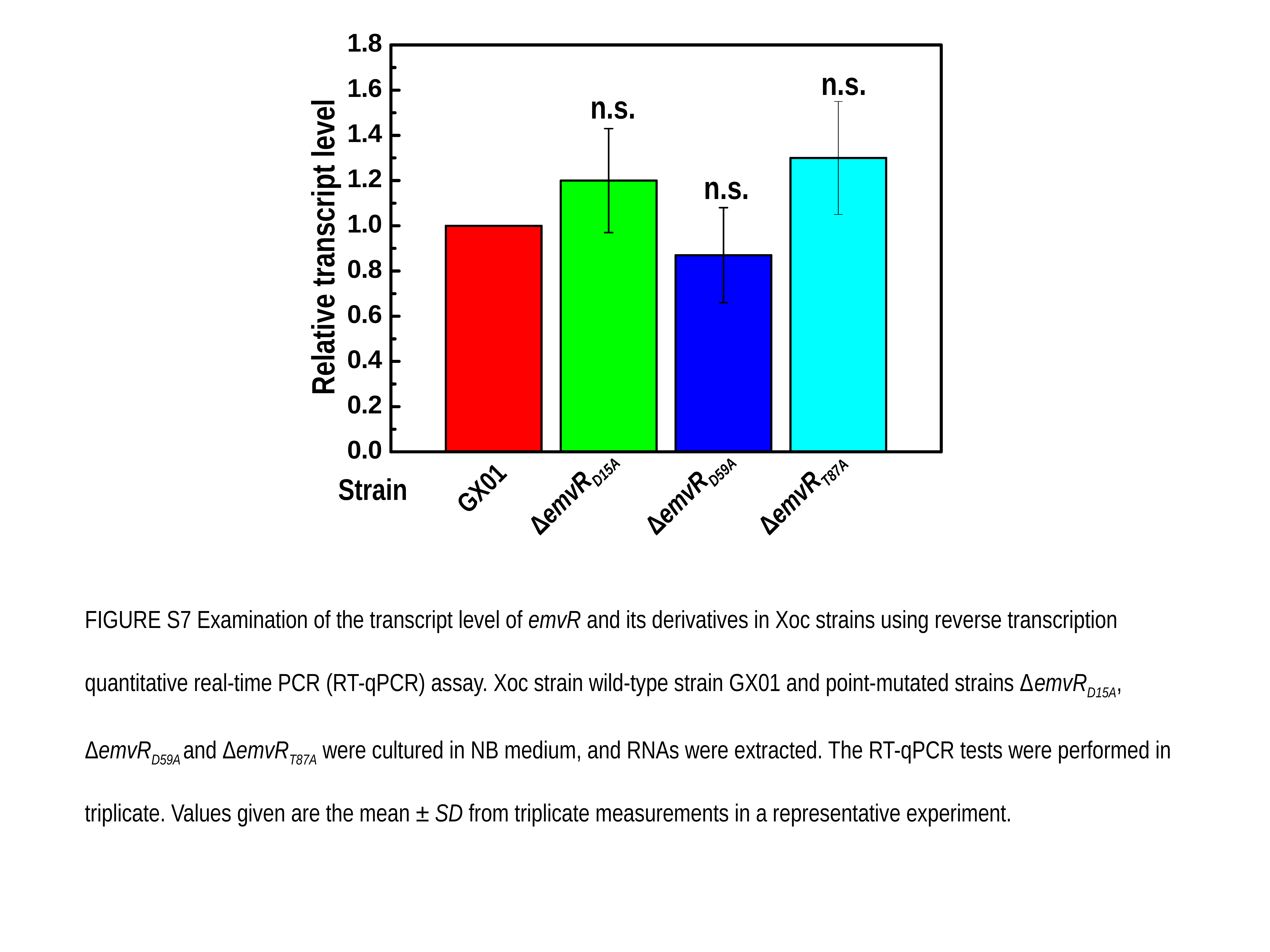

Strain
GX01
ΔemvRD15A
ΔemvRD59A
ΔemvRT87A
FIGURE S7 Examination of the transcript level of emvR and its derivatives in Xoc strains using reverse transcription quantitative real-time PCR (RT-qPCR) assay. Xoc strain wild-type strain GX01 and point-mutated strains ΔemvRD15A, ΔemvRD59A and ΔemvRT87A were cultured in NB medium, and RNAs were extracted. The RT-qPCR tests were performed in triplicate. Values given are the mean ± SD from triplicate measurements in a representative experiment.
