# Supplementary material for: Xanthomonas oryzae Orphan Response Regulator EmvR Is Involved in Virulence, Extracellular Polysaccharide Production and Cell Motility
Source: Mol Plant Pathol. 2025 Apr 6;26(4):e70083. doi: 10.1111/mpp.70083 (PMC11973254; doi:10.1111/mpp.70083)
Supplement: Supplementary file 8 — Table S1. Bacterial strains and plasmids used in this work. [file MPP-26-e70083-s004.docx]

**Table S1**. Bacterial strains and plasmids used in this work

| **Strains or plasmids** | **Relevant characteristics** | **Reference or source** |
| --- | --- | --- |
| ***E. coli* strains** |  |  |
| DH5α | Φ80△*lacZM*15 *recA1 endA1 deoR* | Gibco BRL, Life Technologies |
| BL21(DE3) | F^-^ *ompT gal dcm lon hsdS_B_* (*r^-^_B_ m^-^_B_*) λ(DE3) | Novagen, Germany |
| XL1-Blue MRF' | Reporter strain, Δ*(mcrA)183* Δ*(mcrCB-hsdSMR-mrr)173 endA1 hisB supE44 thi-1 recA1 gyrA96relA1 lac* [F*´ lacIq HIS3 aadA* Kan*^r^*] | Stratagene |
| ***Xanthomonas oryzae* pv. *oryzicola* strains** |  |  |
| GX01 | Wild-type strain, isolated from Guangxi, China, Rif^r^ | Author’s lab collection |
| Δ*emvR* | As GX01, but *XOCgx_1445* (*emvR*) gene deleted, non-polar effect. Rif^r^ | This work |
| CΔ*emvR* | Δ*emvR* harboring the recombinant plasmid pXC*emvR.* Rif^r^ Kan^r^ | This work |
| GX01/pX*emvR* | GX01 harboring the recombinant plasmid pXC*emvR*. Rif^r^ Kan^r^ | This work |
| GX01/pXUK | GX01 harboring the empty vector pXUK. Rif^r^ | This work |
| Δ*colS_XOCgx_4036_* | As GX01, but *colS_XOCgx_4036_* gene deleted, non-polar effect. Rif^r^ | This work |
| CΔ*colS_XOCgx_4036_* | Δ*colS_XOCgx_4036_* harboring the recombinant plasmid pXC *colS_XOCgx_4036_* Rif^r^ Kan^r^ | This work |
| Δ*colR_XOCgx_4037_* | As GX01, but *colR_XOCgx_4037_* gene deleted, non-polar effect. Rif^r^ | This work |
| CΔ*colR_XOCgx_4037_* | Δ*colR_XOCgx_4037_* harboring the recombinant plasmid pXC *colR_XOCgx_4037_* Rif^r^ Kan^r^ | This work |
| Δ*emvR*/pXC*colS_XOCgx_4036_* | Δ*emvR* harboring the recombinant plasmid pLC/pXC*colS_XOCgx_4036_*. Rif^r^ Kan^r^ | This work |
| Δ*emvR*/pXC*colR_XOCgx_4037_* | Δ*emvR* harboring the recombinant plasmid pLC/pXC*colR_XOCgx_4037_*. Rif^r^ Kan^r^ | This work |
| ∆*colS_XOCgx_4036_*/pXC*emvR* | ∆*colS_XOCgx_4036_* harboring the recombinant plasmid pXC*emvR*. Rif^r^ Kan^r^ | This work |
| ∆*colR_XOCgx_4037_*/pXC*emvR* | ∆*colR_XOCgx_4037_* harboring the recombinant plasmid pXC*emvR*. Rif^r^ Kan^r^ | This work |
| Δ*pilB* | As GX01, but *XOCgx_1260* (*pilB*) gene deleted, non-polar effect. Rif^r^ | This work |
| CΔ*pilB* | Δ*pilB* harboring the recombinant plasmid pXC*pilB.* Rif^r^ Kan^r^ | This work |
| GX01(EmvR::3×Flag) | As GX01, but chromosomally encoding a 3×Flag fused EmvR protein. Rif^r^ | This work |
| Δ*emvR_D15A_* | Strain GX01 derivative with *emvR* replaced *in situ* by *emvR_D15A_* | This work |
| Δ*emvR_D59A_* | Strain GX01 derivative with *emvR* replaced *in situ* by *emvR_D59A_* | This work |
| Δ*emvR_T87A_* | Strain GX01 derivative with *emvR* replaced *in situ* by *emvR_T87A_* | This work |
| **Plasmids** |  |  |
| pXUK | Broad host range cloning vector with *lacZ* promoter. Kan^r^ | Authors’ lab collection |
| pXC*emvR* | pXUK containing an 432-bp DNA fragment containing *emvR* gene (*XOCgx_1445*) of *Xoc* strain. Tet^r^ | This work |
| pXC*colR_XOCgx_4037_* | pXUK containing an 747-bp DNA fragment of the ORF *colR_XOCgx_4037_* of *Xoc* strain. Tet^r^ | This work |
| pXC*colS_XOCgx_4036_* | pXUK containing an 822-bp DNA fragment of the ORF *colS_XOCgx_4036_* of *Xoc* strain. Tet^r^ | This work |
| pXC*pilB* | pXUK containing an 1734-bp DNA fragment of the ORF *pilB* of *Xoc* strain. Tet^r^ | This work |
| pRK2073 | Helper plasmid, Tra^+^, Mob^+^, ColE1, Spc^r^. | Leong *et al*.,1982 |
| pK18*mob* | pUC18 derivative, *lacZα* Kan^r^, *mob* site. Suicide plasmid in *Xoc*. | Schäfer *et al.*, 1994 |
| pK*emvR* | pK18*mob* harboring an 432-bp DNA fragment containing *emvR* gene. Kan^r^ | This work |
| pK18*mobsacB* | pUC18 derivative, *lacZα*, *sacB*, Kan^r^, *mob* site. Allelic exchange vector (Suicidal vector carrying *sacB* gene for mutagenesis). | Schäfer *et al*., 1994 |
| pKΔ*emvR* | pK18*mobsacB* containing fragments flanking *emvR* gene. Kan^r^ | This work |
| pKΔ*colR_XOCgx_4037_* | pK18*mobsacB* containing fragments flanking *colR_XOCgx_4037_* gene. Kan^r^ | This work |
| pKΔ*colS_XOCgx_4036_* | pK18*mobsacB* containing fragments flanking *colS_XOCgx_4036_* gene. Kan^r^ | This work |
| pKΔ*pilB* | pK18*mobsacB* containing fragments flanking *pilB* gene. Kan^r^ | This work |
| pK*emvR*::flag | pK18*mobsacB* containing fragment composing 96-bp upstream of *emvR*, 402-bp EmvR-coding sequence, 66-bp 3×Flag-coding sequence, 3-bp stop codon and 500-bp downstream of *emvR*. | This work |
| pBT | Two-hybrid system bait plasmid containing the *cat* gene, p15A origin of replication and λ cI ORF. | Stratagene |
| pBT*emvR* | pBT derivative carrying a 402-bp fragment of *emvR* gene. Cat^r^ | This work |
| pTRG | Two-hybrid system target plasmid containing the *tet* gene, ColE1 origin of replication, and RNA polymerase α subunit ORF. | Stratagene |
| pTRG*pilO* | pTRG derivative carrying 666-bp fragment of *pilO* gene coding region. Tet^r^ | This work |
| pTRG*pilB* | pTRG derivative carrying 1734-bp fragment of *pilB* gene coding region. Tet^r^ | This work |
| pTRG*pilU* | pTRG derivative carrying 1131-bp fragment of *pilU* gene coding region. Tet^r^ | This work |
| pTRG*pilT* | pTRG derivative carrying 1035-bp fragment of *pilT* gene coding region. Tet^r^ | This work |
| pTRG*colS* | pTRG derivative carrying 822-bp fragment of *colS_XOCgx_4036_* gene sequence. Tet^r^ | This work |
| pTRG0533 | pTRG derivative carrying 1971-bp fragment of *XOCgx_0533* gene coding region. Tetr | This work |
| pTRG1563 | pTRG derivative carrying 429-bp fragment of *XOCgx_1563* gene coding region. Tet^r^ | This work |
| pTRG*2122* | pTRG derivative carrying 180-bp fragment of *XOCgx_2212* (*mcp*) gene coding region. Tet^r^ | This work |
| pTRG*2478* | pTRG derivative carrying 1032-bp fragment of *XOCgx_2478* (*mcp*) gene coding region. Tet^r^ | This work |
| pTRG*2601* | pTRG derivative carrying 1758-bp fragment of *XOCgx_2601* (*mcp*) gene coding region. Tet^r^ | This work |
| pTRG*2603* | pTRG derivative carrying 1623-bp fragment of *XOCgx_2603* (*mcp*) gene coding region. Tet^r^ | This work |
| pTRG*2604* | pTRG derivative carrying 1608-bp fragment of *XOCgx_2604* (*mcp*) gene coding region. Tet^r^ | This work |
| pTRG*2606* | pTRG derivative carrying 2322-bp fragment of *XOCgx_2606* (*mcp*) gene coding region. Tet^r^ | This work |
| pTRG*2861* | pTRG derivative carrying 1572-bp fragment of *XOCgx_2861* (*mcp*) gene coding region. Tet^r^ | This work |
| pET-30a | Expression vector, allow the production of fusion proteins containing amino terminal 6×His-tagged sequences. Kan^r^ | Novagen |
| pET-30a-EmvR | pET-30a cloned into a 402-bp DNA fragment of the *emvR* gene coding sequence | This work |
| pET-30a-ColS_XOCgx_4036_ | pET-30a cloned into a 822-bp DNA fragment of the *colS_XOCgx_4036_* gene coding sequence | This work |
| pET-30a-PilB | pET-30a cloned into a 1734-bp DNA fragment of the *pilB* gene coding sequence | This work |
| pET-30a-PilO | pET-30a cloned into a 666-bp DNA fragment of the *pilO* gene coding sequence | This work |
| pET-30a-PilU | pET-30a cloned into a 1131-bp DNA fragment of the *pilU* gene coding sequence | This work |
| pET-30a-PilT | pET-30a cloned into a 1035-bp DNA fragment of the *pilT* gene coding sequence | This work |

^a^Rif^r^, Kan^r^, Tet^r^, Ampr and Spc^r^ indicate resistance to rifampicin, kanamycin, tetracycline, ampicillin and spectinomycin, respectively.

**References**

Leong, S.A., Ditta, G.S., and Helinski, D.R. (1982) Heme biosynthesis in *Rhizobium*. Identification of a cloned gene coding for delta-aminolevulinic acid synthetase from *Rhizobium meliloti*. *J. Biol. Chem.* 257, 8724–8730.

Schäfer, A., Tauch, A., Jäger, W., Kalinowski, J., Thierbach, G., and Pühler, A. (1994) Small mobilizable multi-purpose cloning vectors derived from the *Escherichia coli* plasmids pK18 and pK19: selection of defined deletions in the chromosome of *Corynebacterium glutamicum*. *Gene* 145, 69–73.
