# Supplementary material for: Xanthomonas oryzae Orphan Response Regulator EmvR Is Involved in Virulence, Extracellular Polysaccharide Production and Cell Motility
Source: Mol Plant Pathol. 2025 Apr 6;26(4):e70083. doi: 10.1111/mpp.70083 (PMC11973254; doi:10.1111/mpp.70083)
Supplement: Supplementary file 9 — Table S2. The potential EmvR‐interacting proteins identified by co‐immunoprecipitation coupled with liquid chromatography–tandem‐mass spectrometry (LC–MS/MS) assays. [file MPP-26-e70083-s008.docx]

**Table S2.** The potential EmvR-interacting proteins identified by Co-IP coupled with LC-MS/MS assays

| **Gene ID** | **Name** | **Annotation** |
| --- | --- | --- |
| *XOCgx_0533* |  | Two-component system response regulator protein with GGDEF and EAL |
| *XOCgx_0821* | *atpG* | ATP synthase gamma chain |
| *XOCgx_0851* | *fabA* | 3-hydroxydecanoyl-[acyl-carrier-protein] dehydratase |
| *XOCgx_0890* | *rfbA* | Glucose-1-phosphate thymidylyltransferase |
| *XOCgx_1015* |  | L-isoaspartyl protein carboxyl methyltransferase |
| *XOCgx_1113* | *pilO* | Fimbrial protein |
| *XOCgx_1116* | *moxR* | ATPase AAA |
| *XOCgx_1260* | *pilB* | Fimbrial protein/Secretion pathway protein GspE |
| *XOCgx_1563* |  | Histidine kinase |
| *XOCgx_1719* | *bamA* | Outer membrane protein assembly factor BamA |
| *XOCgx_1859* | *nuoC* | NADH-quinone oxidoreductase subunit C |
| *XOCgx_1862* | *nuoF* | NADH-quinone oxidoreductase subunit F |
| *XOCgx_1863* | *ACU17_08220* | NADH-quinone oxidoreductase |
| *XOCgx_1866* |  | NADH-quinone oxidoreductase subunit J |
| *XOCgx_2075* | *hflB* | ATP-dependent zinc metalloprotease FtsH |
| *XOCgx_2212* | *mcp* | Methyl-accepting chemotaxis protein |
| *XOCgx_2276* | *ugpC* | Glycerol-3-phosphate transporter ATP-binding subunit |
| *XOCgx_2478* | *mcp* | Methyl-accepting chemotaxis protein |
| *XOCgx_2510* | *flrA* | Fis family transcriptional regulator |
| *XOCgx_2601* | *mcp* | Methyl-accepting chemotaxis protein |
| *XOCgx_2603* | *mcp* | Methyl-accepting chemotaxis protein |
| *XOCgx_2604* | *mcp* | Methyl-accepting chemotaxis protein |
| *XOCgx_2606* | *mcp* | Methyl-accepting chemotaxis protein |
| *XOCgx_2642* | *carB* | Carbamoyl-phosphate synthase large chain |
| *XOCgx_2861* | *mcp* | Methyl-accepting chemotaxis protein |
| *XOCgx_2881* | *phoH* | phoH |
| *XOCgx_2935* |  | Glutamate dehydrogenase |
| *XOCgx_2943* | *mtp* | EIII-Fru |
| *XOCgx_2959* | *secD* | Protein translocase subunit SecD |
| *XOCgx_3167* |  | Transcriptional regulator |
| *XOCgx_3448* | *pilU* | Fimbrial protein |
| *XOCgx_4036* | *colS* | Two-component system sensor kinase |
| *XOCgx_4128* | *ribH* | 6,7-dimethyl-8-ribityllumazine synthase |
| *XOCgx_4129* | *ribB* | 3,4-dihydroxy-2-butanone 4-phosphate synthase |
| *XOCgx_4253* | *hslU* | ATP-dependent protease ATPase subunit HslU |
