# Supplementary material for: Xanthomonas oryzae Orphan Response Regulator EmvR Is Involved in Virulence, Extracellular Polysaccharide Production and Cell Motility
Source: Mol Plant Pathol. 2025 Apr 6;26(4):e70083. doi: 10.1111/mpp.70083 (PMC11973254; doi:10.1111/mpp.70083)
Supplement: Supplementary file 10 — Table S3. Primers used in this work. [file MPP-26-e70083-s010.docx]

**Table S3. Primers used in this study^§^**

| Primer | Nucleotide sequence (5′→3′) | The amplified fragment or the utilization |
| --- | --- | --- |
| L*emvR-*F  L*emvR-*R | CGGGATCC TGTCCTGGTCTCTGTGTT  GCTCTAGA TCAGGCCGCATTCTCAGC | 543-bp DNA sequence upstream of *emvR* (*XOCgx_1445*), used for constructing the *emvR* deletion mutant. |
| R*emvR-*F  R*emvR-*R | GCTCTAGA GCCGGTCGCGTAACACTG  CCAAGCTT AGATCAGGACGCAGGTCC | 526-bp DNA sequence downstream of *emvR*, used for constructing the *emvR* deletion mutant. |
| *emvR-*OF  *emvR*-OR | CGGGATCCATGCGGCCTGATTTACGAGACC  CCAAGCTTTTACGCGACCGGCGTGGCTGGC | 432-bp DNA fragment containing the *emvR* coding sequence. Cloned into the vector pXUK for complementation or expression vector pET-30a for protein overproduction and pull-down assays. |
| D15-F  D15-R | ATTCGTTGTCGAAGCCGGCGATGGCACCCGACA  CGGCTTCGACAACGAATACAACCGGCCGTTCC | Used for *emvR* site-directed mutagenesis, replacing Asp15 to Ala15. |
| D59-F  D59-R | GTTGTTTTCCGCTATCCATTTCCCGGGCGGTC  GGATAGCGGAAAACAACACGTCGACGCGTGGA | Used for *emvR* site-directed mutagenesis, replacing Asp59 to Ala59. |
| T87-F  T87-R | TCCTGCTGGCCTCCGGCCTGGCGGTCGAGTAC  GCCGGAGGCCAGCAGGACGGGCAGGTTATACG | Used for emvR site-directed mutagenesis, replacing Thr87 to Ala87. |
| L*colS-*F  L*colS-*R | CGGGATCCCTGACCAAGCCGTTTGCGATTCA  GCTCTAGATGTGGCATTGCTGCATCCAGGAG | 543-bp DNA sequence upstream of *colS_XOCgx_4036_*, used for constructing the *colS_XOCgx_4036_* deletion mutant. |
| R*colS-*F  R*colS-*R | GCTCTAGAGGTAGCGGTCGGTAGCAGGGCGT  CCAAGCTTACCGGTACTGGAAGAACGCTGAA | 595-bp DNA sequence downstream of *colS_XOCgx_4036_*, used for constructing the *colS_XOCgx_4036_* deletion mutant. |
| *colS-*OF  *colS*-OR | CCAAGCTTGAATCGCCGCGCGTGGTGACGC  GCTCTAGATCAGGCCACCACCACCGCTACT | 1609-bp DNA fragment of the *colS_XOCgx_4036_* coding sequence. Cloned into the vector pXUK for complementation |
| *colS-*pOF  *colS*-pOR | CGGGATCCCGTGTCTCCAAACGGCTGGTCTCG  CCAAGCTTTCAGGCCACCACCACCGCTACTGA | 822-bp DNA fragment of truncated *colS_XOCgx_4036_* coding sequence. Cloned into the expression vector pET-30a for protein overproduction and pull-down assays. |
| L*colR-*F  L*colR-*R | CGGGATCCCAATGCCAGCATCAGCGGCGAGA  GCTCTAGATTCAGCTTTGTGAAACATGCCGG | 506-bp DNA sequence upstream of *colR_XOCgx_4037_*, used for constructing the *colR_XOCgx_4037_* deletion mutant. |
| R*colR-*F  R*colR-*R | GCTCTAGACCAACCGATGGCCTGATGTCCT  CCAAGCTTTCGACATACACCAGCGCATCGG | 478-bp DNA sequence downstream of *colR_XOCgx_4037_*, used for constructing the *colR_XOCgx_4037_* deletion mutant. |
| *colR-*OF  *colR*-OR | CCAAGCTTGGCCTGCTGGAAAAACACCATCCC  GCTCTAGATCAGGCCATCGGTTGGGCGATGT | 1183-bp DNA fragment of the *colR_XOCgx_4037_* coding sequence. Cloned into the vector pXUK for complementation |
| *pilT-*OF  *pilT-*OR | CGGGATCCATGGATATCGCTGAACTA  CCCTCGAGCTATTCGAATATCCGCTT | 1035-bp DNA fragment of the *pilT* (*XOCgx_3449* ) coding sequence. Clone into target vector pTRG for bacterial two-hybrid assays, or expression vector pET-30a for protein overproduction and pull-down assays |
| L*pilB-*F  L*pilB-*R | CGGGATCC CACATCCATATCGACGAC  GCTCTAGA CATCGTTTGATTCCCGCT | 536-bp DNA sequence upstream of *pilB* (*XOCgx_1260*), used for constructing the *pilB* deletion mutant. |
| R*pilB-*F  R*pilB-*R | GCTCTAGA ATCGGGTGACCAAGGACT  CCCAAGCTT AGTCGACCAGGATATGTT | 552-bp DNA sequence downstream of *pilB*, used for constructing the *pilB* deletion mutant. |
| C*pilB-*OF  C*pilB-*OR | CCCAAGCTTGATGAATTCAGTATCTACAACC  GCTCTAGATCAGTCCTTGGTCACCCG | 1734-bp DNA fragment of *pilB* coding sequence. Clone into the vector pXUK for complementation. |
| *pilB-*OF  *pilB-*OR | GGGGATCCATGAATTCAGTATCTACAACCAACCTCG  CCAAGCTTTCAGTCCTTGGTCACCCGATTGAT | 1734-bp DNA fragment of *pilB* coding sequence. Clone into expression vector pET-30a for protein overproduction and pull-down assays. |
| *pilO-*OF  *pilO-*OR | CGGAATTCAGATGAGTAAGAAATCATTCAAGCTC  CCCTCGAGTCATTGGCCGCCCTTCTTCGCA | 666-bp DNA fragment of the *pilO* sequence. Cloned into target vector pTRG for bacterial two-hybrid assays, or into expression vector pET-30a for protein overproduction and pull-down assays. |
| *pilU-*OF  *pilU-*OR | CGGGATCCATGAGCACCATCGACTTCACCTCC  CCCTCGAGTTATCGAACTTCGGAAATCTCCACACC | 1131-bp DNA fragment of the *pilU* sequence. Cloned into target vector pTRG for bacterial two-hybrid assays, or into expression vector pET-30a for protein overproduction and pull-down assays. |
| L*emvR-*FlagF  L*emvR-*FlagR | CGGGATCCGGCCCATTCGGCTGGTAACCAT  TGTCATGATCTTTATAATCACCGTCATGGTCTTTGTAGTCCGCGACCGGCGTGGCTGGC | 538-bp DNA fragment containing 96-bp DNA upstream of the *emvR*, 402-bp EmvR-coding sequence, and 40-bp Flag-coding sequence. Used for constructing *Xoc* strain producing EmvR::3×Flag protein. |
| R*emvR-*FlagF  R*emvR-*FlagR | TGATTATAAAGATCATGACATCGACTACAAGGATGACGATGACAAGTAACACTGGCGGCCGCGAC  CCAAGCTTGGTCCTCCGAACATCGAACCCG | 549-bp DNA fragment containing 46-bp Flag-coding sequence, 3-bp stop codon and 500-bp downstream of the *emvR* stop codon. Used for constructing *Xoc* strain producing Emv::3×Flag protein. |
| *emvR*-BTF  *emvR*-BTR | CGGAATTCCATGCCACAAAACAAGGAACGGCC  CGGGATCCTTACGCGACCGGCGTGGCTG | 402-bp DNA fragment of the *emvR* coding sequence. Cloned into bait vector pBT for bacterial two-hybrid assays, or expression vector pET-30a for protein overproduction. |
| *colS*-TRGF  *colS*-TRGR | CGGGATCCCGTGTCTCCAAACGGCTGGTCTCG  CCCTCGAGTCAGGCCACCACCACCGCTACTGA | 822-bp DNA fragment of the *colS_XOCgx_4036_* sequence. Cloned into target vector pTRG for bacterial two-hybrid assays. |
| *colR*-TRGF  *colR*-TRGR | CGGGATCCATGTTTCACAAAGCTGAAGACCG  CCCTCGAGTCAGGCCATCGGTTGGGCGATGT | 747-bp DNA fragment of the *colR_XOCgx_4037_* sequence. Cloned into target vector pTRG for bacterial two-hybrid assays. |
| *pilB*-TRGF  *pilB*-TRGR | CCCTCGAGATGAATTCAGTATCTACAACCAACC  GGACTAGTTCAGTCCTTGGTCACCCGATTGAT | 1734-bp DNA fragment of the *pilB* sequence. Cloned into target vector pTRG for bacterial two-hybrid assays. |
| 0533-TRGF  0533-TRGR | CGGGATCCATGGCCGCCCGAGATCCGGTT  CCCTCGAG TCAGAGCACCGCGTTCGTGAA | 1971-bp DNA fragment of the *XOCgx_0533* sequence. Cloned into target vector pTRG for bacterial two-hybrid assays. |
| 1563-TRGF  1563-TRGR | CGGGATCCATGCAGATCGTACGACAGCTGTTG  CCCTCGAGTCAACTGGCGATGTAGCGCTGCAG | 429-bp DNA fragment of the *XOCgx_1563* sequence. Cloned into target vector pTRG for bacterial two-hybrid assays. |
| 2212-TRGF  2212-TRGR | CGGGATCCAACAGAGTGTTGCGCCCCGTTC  CCCTCGAGTCATCGTTTGTTGAGCCGCTCC | 180-bp DNA fragment of the *XOCgx_2212* (*mcp*) sequence. Cloned into target vector pTRG for bacterial two-hybrid assays. |
| 2478-TRGF  2478-TRGR | CGGGATCCCAGTTCCGGTCTAACCCGGACC  CGGAATTCTTAGAACAACTCGACCGTGCCG | 1032-bp DNA fragment of the *XOCgx_2478* (*mcp*) sequence. Cloned into target vector pTRG for bacterial two-hybrid assays. |
| 2601-TRGF  2601-TRGR | CGGGATCCCTGCGTCGCCAGGTGGTGCAGC  CCCTCGAGTCAGACGTCCTGCCACTGGCTG | 1758-bp DNA fragment of the *XOCgx_2601* (*mcp*) sequence. Cloned into target vector pTRG for bacterial two-hybrid assays. |
| 2603-TRGF  2603-TRGR | CGGGATCCACCCGCAGCCTCACCCAGCCGT  CCCTCGAGCTAGAACTCGGCCCAATTGGAC | 1623-bp DNA fragment of the *XOCgx_2603* (*mcp*) sequence. Cloned into target vector pTRG for bacterial two-hybrid assays. |
| 2604-TRGF  2604-TRGR | CGGGATCCCGCCGTCGTCTGATCAACCGCC  CCCTCGAGTCAGAACTCCTGCCAGCTGGTC | 1608-bp DNA fragment of the *XOCgx_2604* (*mcp*) sequence. Cloned into target vector pTRG for bacterial two-hybrid assays. |
| 2606-TRGF  2606-TRGR | CGGGATCCGGCAAGCTCAACGAAAGCGTCG  CCCTCGAGTCAGGCAGCGCGACGCAGGCCG | 2322-bp DNA fragment of the *XOCgx_2606* (*mcp*) sequence. Cloned into target vector pTRG for bacterial two-hybrid assays. |
| 2861-TRGF  2861-TRGR | CGGGATCCTATTCCAACTCCGGCCGCATCC  CCCTCGAGTCAGAAACGCGCGAACTGGCCT | 1572-bp DNA fragment of the *XOCgx_2861* (*mcp*) sequence. Cloned into target vector pTRG for bacterial two-hybrid assays. |
| 16SF  16SR | GCCTAACACATGCAAGTCGAACGGC  AATATTCCCCACTGCTGCCTCCCG | 325-bp DNA fragment of the 16S rDNA sequence, used for RT-PCR and qRT-PCR. |
| *gumB* -F  *gumB* -R | ACGATTTTGCCAAGCGCTG  TGGCCGTTCTGATCGATACG | 219-bp DNA fragment spans nucleotides 18 to 236 bp of the *gumB* (*XOCgx_3054*), used for RT-PCR. |
| *gumD*-F  *gumD*-R | TGCGGTCCATGCGTTGATC  CATACCAACCCACGAGTTGCG | 232-bp DNA fragment spans nucleotides 318 to 549 bp of the *gumD*, used for qRT-PCR. |
| *gumF*-F  *gumF*-R | CGTAATGTCGGTGAGAAGGCTG  GGGAACCAGTTCATCCACAACC | 230-bp DNA fragment spans nucleotides 340 to 569 bp of the *gumF*, used for qRT-PCR. |
| 2502-F  2502-R | CGCCAACATCGATACGCTGG  TGATCGCCGAGGCAACCTTC | 215-bp DNA fragment spans nucleotides 492 to 706 bp of the *fliC* (*XOCgx_2502*), used for qRT-PCR. |
| 2496-F  2496-R | TTGTGGGTCGCCAAAACCG  AGCCCTTGAACGTGGACACC | 229-bp DNA fragment spans nucleotides 13 to 241 bp of the *flgG* (*XOCgx_2496*), used for qRT-PCR. |
| 2540-F  2540-R | GCCCTGACTGAATTGCGTGG  TGAACTCCACCAGATGCCCC | 214-bp DNA fragment spans nucleotides from 16 to 229 bp of the *fliQ* (*XOCgx_2540*), used for qRT-PCR. |
| 2536-F  2536-R | GAACGCCGCCGACCTTAATC  CCGAAGCGGTCGTTGATGAC | 216-bp DNA fragment spans nucleotides 69 to 284 bp of the *fliN* (*XOCgx_2536*), used for qRT-PCR. |
| 1260-F  1260-R | CGACGCTAACCAGAACGCAG  CCACTGCTCCAATGTGCGAC | 214-bp DNA fragment spans nucleotides 231 to 444 bp of the *pilB* (*XOCgx_1260*), used for qRT-PCR. |
| 1480-F  1480-R | GTTGTTGCCGCTGCTCAATG  TCTTTGACGCGCAGCAATTC | 204-bp DNA fragment spans nucleotides 357 to 560 bp of the *pilL* (*XOCgx_1480*), used for qRT-PCR. |
| 1113-F  1113-R | CGAAGCAGGCACGTATCGTG  ACCTGTTCCATCTGCGCGAG | 205-bp DNA fragment spans nucleotides 59 to 263 bp of the *pilO* (*XOCgx_1113*), used for qRT-PCR. |
| 3448-F  3448-R | AGAGCCGCGATCTGGTGTTG  TCTTGATCACCGGAGGCAGG | 213-bp DNA fragment spans nucleotides 146 to 358 bp of the *pilU* (*XOCgx_3448*), used for qRT-PCR. |
| 2651-F  2651-R | TGTGCCGTGGCTTCTATCCG  ACTGCCACCGTTTCGACCAC | 229-bp DNA fragment spans nucleotides 242 to 470 bp of the *XOCgx_2651* (*feoB*), used for RT-PCR. |
| 2201-F  2201-R | GAAGCCGCATCAGGAAGACC  TTCGCGCCCGCTATCTGTAC | 208-bp DNA fragment spans nucleotides 6 to 213 bp of the *XOCgx_2201*, used for RT-PCR. |
| 1460-F  1460-R | TTACTCGCGATAGCCAGCCG  TTCGATCACGTCGGCTTCG | 227-bp DNA fragment spans nucleotides 11 to 237 bp of the *XOCgx_1460*, used for RT-PCR. |
| 2202-F  2202-R | TCCTGCATGTCAATGCCCTG  GTATTCAATCGTGGCGCTCG | 212-bp DNA fragment spans nucleotides 236 to 447 bp of the *XOCgx_2202*, used for RT-PCR. |
| 1508-F  1508-R | TGGCTTTTCCAACCGCAGTC  ATCACGCCCTGGTTGTTGG | 205-bp DNA fragment spans nucleotides 2 to 206 bp of the *XOCgx_1508*, used for RT-PCR. |
| 0933-F  0933-R | TCGATAAGCTGGCAGGAACG  GGAAATCGGGAATCGGGAG | 161-bp DNA fragment spans nucleotides 5 to 165 bp of the *XOCgx_0933*, used for RT-PCR. |
| 2704-F  2704-R | GCTCGGTTATGCATTGGACG  AAGCCAAACGGCAATGCC | 180-bp DNA fragment spans nucleotides 114 to 293 bp of the *XOCgx_2704*, used for RT-PCR |
| 2623-F  2623-R | GACCTGATGGAAACCCTGGC  AATCGCGGAAGAAGCCCTC | 205-bp DNA fragment spans nucleotides163 to 367 bp of the *XOCgx_2623*, used for RT-PCR. |
| 3758-F  3758-R | CGATCAACGCCAACCATGC  TCGATGTTGAACGCGCTCG | 220-bp DNA fragment spans nucleotides 83 to 302 bp of the *XOCgx_3758*, used for RT-PCR. |
| 2239-F  2239-R | ATGATGACAATCACCGCG  TCATGTGGGTGACTCCGA | 144-bp DNA fragment spans nucleotides 1 to 144 bp of the *XOCgx_2239*, used for qRT-PCR. |

^§^The underlined sequences indicate the restriction sites for *Bam*HI, *Eco*RI, *Hin*dIII, *Kpn*I, *Pst*I, *Sac*I, *Xba*I and *Xho*I, respectively. The long square boxes indicate the Flag-coding sequences, and highlights in gray show the complementary sequences.
